# Genetic variation in ABCB5 associates with risk of hepatocellular carcinoma

**DOI:** 10.1111/jcmm.15691

**Published:** 2020-08-11

**Authors:** Idy C.‐Y. Leung, Charing C.‐N. Chong, Tan T. Cheung, Philip C. Yeung, Kelvin K.‐C. Ng, Paul B.‐S. Lai, Stephen L. Chan, Anthony W.‐H. Chan, Patrick M.‐K. Tang, Siu T. Cheung

**Affiliations:** ^1^ Department of Surgery The University of Hong Kong Hong Kong Hong Kong; ^2^ Department of Surgery The Chinese University of Hong Kong Hong Kong Hong Kong; ^3^ State Key Laboratory of Translational Oncology Department of Clinical Oncology Sir YK Pao Centre for Cancer The Chinese University of Hong Kong Hong Kong Hong Kong; ^4^ Department of Anatomical and Cellular Pathology State Key Laboratory of Translational Oncology The Chinese University of Hong Kong Hong Kong Hong Kong; ^5^ Li Ka Shing Institute of Health Sciences Prince of Wales Hospital The Chinese University of Hong Kong Hong Kong Hong Kong

**Keywords:** drug transporter, liver cancer, multidrug resistance

## Abstract

Expression of ATP‐binding cassette B5 (ABCB5) has been demonstrated to confer chemoresistance, enhance cancer stem cell properties and associate with poor prognosis in hepatocellular carcinoma (HCC). The aim of this study was to evaluate the genetic variations of ABCB5 in HCC patients with reference to healthy individuals and the clinicopathological significance. A pilot study has examined 20 out of 300 pairs HCC and paralleled blood samples using conventional sequencing method to cover all exons and exon/intron regions to investigate whether there will be novel variant sequence and mutation event. A total of 300 HCC and 300 healthy blood DNA samples were then examined by Sequenom MassARRAY genotyping and pyrosequencing for 38 SNP and 1 INDEL in ABCB5. Five novel SNPs were identified in ABCB5. Comparison of DNA from blood samples of HCC and healthy demonstrated that ABCB5 SNPs rs75494098, rs4721940 and rs10254317 were associated with HCC risk. Specific ABCB5 variants were associated with aggressive HCC features. SNP rs17143212 was significantly associated with ABCB5 expression level. Nonetheless, the paralleled blood and tumour DNA sequences from HCC patients indicated that ABCB5 mutation in tumours was not common and corroborated the TCGA data sets. In conclusion, ABCB5 genetic variants had significant association with HCC risk and aggressive tumour properties.

## 1 INTRODUCTION

ATP‐binding cassette (ABC) transporter superfamily is a large group of transporters across cell membranes and organelle membranes.^1^ ABC transporters convey a large spectrum of molecules by utilizing the energy generated by hydrolysis of ATP against their electrochemical gradient.^1^, ^2^ ABC transporters are expressed in different organs and tissues. They play a pivotal role in maintaining homeostasis, protecting against toxin as well as excretory and secretory functioning.^3^, ^4^ Many ABC transporters also confer multidrug resistance (MDR) by effluxing a broad‐spectrum of chemotherapeutic agents to avoid their intracellular accumulation.^5^ This largely reduces the cytotoxicity of the chemotherapeutic agents, thereby affecting the drug sensitivity and response, and resulting in recurrence as well as death.^5^, ^6^

In recent decades, extensive studies revealed that MDR in hepatocellular carcinoma (HCC) was mediated by overexpression of ABC transporters including ABCB1, ABCB5, ABCC1, ABCC2 and ABCG2.^7^, ^8^, ^9^, ^10^ Overexpression of these ABC transporters was observed in cancer stem cells (CSC) with enhanced stem cell properties such as self‐protection by resistance to drug, self‐renewal, differentiation and proliferation after a period of quiescent state.^7^, ^11^, ^12^, ^13^ ABCB5 is present on chromosome 7. Deletion of chromosome 7 in HCC was an uncommon event,^14^, ^15^ while chromosomal gains at chromosome 7 were frequently observed in HCC.^16^ In particular, ABCB5 belongs to sub‐family B of the ABC transporter superfamily,^17^ and has been reported to be overexpressed in HCC, associated with chemoresistance, cancer stemness properties and poor recurrence‐free survival.^7^ Interestingly, expression of ABCB5 demonstrated to associate with melanoma initiation, chemoresistance or refractory disease in colorectal cancer and haematological malignancies.^18^, ^19^, ^20^, ^21^, ^22^ Several studies revealed ABCB5 exported doxorubicin, camptothecin, 10‐OH camptothecin, 5‐fluorouracil (5‐FU), paclitaxel and caffeic acid phenethyl ester (CAPE) to confer chemoresistance in subset of cells expressing cancer stem cell phenotypes including melanoma and HCC.^7^, ^18^, ^21^, ^22^, ^23^, ^24^ Interestingly, specific SNPs in ABCB5 have been reported to associate with melanoma risk.^25^ Thus, it would be valuable to examine ABCB5 genetic variation in association with HCC risk. We hypothesized that genetic variations of ABCB5 may alter the gene expression level and recurrence‐free survival of HCC patients. The aim of the present study was to investigate the genetic variants of ABCB5 gene in HCC patients with reference to healthy individuals and to evaluate the significances of genetic variants with respect to clinicopathological features of HCC.

## 2 MATERIALS AND METHODS

### 2.1 Clinical specimens

Clinical samples were collected with informed consent. Majority of HCC patients were Chinese (99.3%, 298/300), and similarly, the majority of the healthy donors were Chinese (94.7%, 284/300). The study was approved by the Institutional Review Board of the University of Hong Kong/ Hospital Authority Hong Kong West Cluster (HKU/HA HKW IRB). Patients underwent hepatectomy for HCC, and healthy individuals were prospectively recruited to the study. All patients had been diagnosed with primary HCC and confirmed by histology. Clinicopathological information including sex, age, tumour size, tumour stage and survival outcomes were collected prospectively. Genomic DNA was extracted from blood sample or tumour tissue by QIAamp DNA Kit (Qiagen, Hilden, Germany). HCC DNA was subjected to DNA sequencing in the pilot Phase 1 study. SNPs were examined by Sequenom MassARRAY system, if sequence not compatible in assay design will be investigated by pyrosequencing, in Phase 2 study comparing HCC and healthy germline genotypes.

### 2.2 DNA sequencing

A total of 28 exons and exon/intron boundaries of ABCB5 gene were examined by polymerase chain reaction (PCR) sequencing with primers and conditions listed in Appendix S1. DNA sequencing was performed by ABI Prism 3730×/ DNA Analyzer (Applied Biosystem, Grand Island, NY, USA) by Tech Dragon Ltd (Hong Kong).

### 2.3 SNP genotyping

Genotyping of SNPs and INDELs were carried out by Sequenom MassARRAY (Sequenom iPLEX Gold Assay, San Diego, CA, USA) by the Centre for Genomic Sciences, the University of Hong Kong. PCR primers and extension primers were designed using Sequenom Mass ARRAY Assay Design 3.1 Software and listed in Appendix S2. The SNPs and INDELs with call rate less than 95% were excluded.

### 2.4 Pyrosequencing

Pyrosequencing of PCR amplification products was carried out by Pyrosequencing System PSQ 96MA (Biotage‐Qiagen, Hilden, Germany) by the Centre for Genomic Sciences, the University of Hong Kong. The PCR and sequencing primers for pyrosequencing were listed in Appendix S3 with biotinylated primers (forward or reverse) (Integrated DNA Technologies, Coralville, Iowa, USA).

### 2.5 Real‐time quantitative RT‐PCR

Quantitative RT‐PCR was performed as described.^7^ Primers and probe for ABCB5 transcript and control 18s were ready‐made reagents (Pre‐Developed TaqMan Assay Reagents; Applied Biosystems). Part of the ABCB5 expression data had been reported previously.^7^

### 2.6 TCGA data

The genomics data and corresponding clinical information for HCC and other cancer types were obtained from The Cancer Genome Atlas (TCGA) (https://cancergenome.nih.gov/). Further clinical data were extracted from cBioPortal (http://www.cbioportal.org/). For samples with duplicate data sets, the average expression values were used.

### 2.7 Sequence alignment across species

SNP and INDEL regions were analysed for sequence variations among different species to understand the evolutionarily change. Multiple sequences were aligned, comparing human (NM_001163941.1) and seven vertebrates including chimpanzee (XM_009452745.1), mouse (NM_029961.2), cow (XM_002686705.3), pig (ENSSSCT00000016752), chicken (ENSGALT00000017732), xenopus (NM_203923.1) and zebrafish (XM_001922682.5). Sequence alignment was performed using Ensembl (http://www.ensembl.org).

### 2.8 Statistics

The association of genotype and HCC risk was evaluated by logistic regression, and the clinicopathological significance was evaluated by chi‐square test and Fisher's exact test for comparison of categorical variable, log‐rank test for comparison of survival outcome and Cox regression for univariate and multivariate analysis of prognostic factors for survival outcome. All analyses were performed with SPSS 25.0 software (IBM Corp., Armonk, NY, USA). *P* value less than .05 was considered significant.

## 3 RESULTS

### 3.1 ABCB5 genetic variant

Genomic DNA was extracted from tumour samples of 20 out of 300 HCC patients, and a total of 28 exons and exon‐intron boundaries of ABCB5 gene were amplified by polymer chain reaction (PCR). A total of 47 single nucleotide polymorphisms (SNPs) and 4 insertions and deletions (INDELs) were identified in ABCB5 gene (Table 1 and Table S1). Forty genetic variants (37 SNPs and 3 INDELs) located at non‐coding region, whereas 11 genetic variants (10 SNPs and 1 INDELs) were in coding region. By comparing to the reported SNPs in the dbSNP database of the National Center for Biotechnology Information (NCBI dbSNP) and published reports, we discovered 5 novel SNPs and submitted them to NCBI dbSNP, including ss1148219560 (−132C>T) in exon 1, ss836312076 (IVS6+17G>T) in intron 6, ss836312077 (IVS14+50T>C) in intron 14, ss836312078 (2166T>G) in exon 18 and ss836312079 (4609A>G) in 3’‐untranslated region of exon 28 could be considered novel (Figure S1). Importantly, all genetic variants identified in the HCC specimens were identical to their parallel blood specimen, indicating that ABCB5 variants were germline mutations.

**TABLE 1:** Identification ABCB5 variations: 47 SNPs and 4 INDELs

Order	dbSNP ID	Nucleotide change^a^	Allele Frequency (%)		Effect	SIFT Score
			Wild‐type allele (Ref. allele)	Variant allele (Alt. allele)		
1	rs79998607	−238A>G	92.5	7.5	Non‐coding	
2	rs2106562	−214C>G	7.5	92.5	Non‐coding	
3	ss1148219560^c^	−132C>T	97.5	2.5	Non‐coding	
4	rs73076550	−90C>T	85	15	Non‐coding	
5	rs57228312	IVS1‐33A>G	92.5	7.5	Non‐coding	
6	rs111872870	IVS2+108C>T	92.5	7.5	Non‐coding	
7	rs75494098	IVS2+135C>T	92.5	7.5	Non‐coding	
8	**rs17143187**	IVS3+1G>C	70	30	**Splicing variant**	
9	rs3033483	IVS3‐75_‐74insTG	85	15	Non‐coding	
10	rs186505679	IVS4‐28T>A	97.5	2.5	Non‐coding	
11	rs76859629	IVS5+46T>G	92.5	7.5	Non‐coding	
12	**rs17143212**	392C>T	92.5	7.5	**Thr‐>Ile**	**0.03**
13	ss836312076^c^	IVS6+17G>T	97.5	2.5	Non‐coding	
14	rs75506657	IVS7+140T>A	97.5	2.5	Non‐coding	
15	rs12669250	IVS7+192A>G	85	15	Non‐coding	
16	**rs2074000**	784C>A	70	30	**Gln‐>Lys**	**0.00**
17	rs11983326	IVS8+5T>G	77.5	22.5	Non‐coding	
18	rs11769236	IVS9+25G>A	92.5	7.5	Non‐coding	
19	rs11772926	IVS9+139T>C	92.5	7.5	Non‐coding	
20	rs2893006	1005C>T	92.5	7.5	Ser‐>Ser	
21	rs75784515	IVS10+61C>T	92.5	7.5	Non‐coding	
22	rs13244876	IVS12+165C>A	82.5	17.5	Non‐coding	
23	rs11973722	IVS12+167T>A	80	20	Non‐coding	
24	rs12674033	IVS12+179G>T	82.5	17.5	Non‐coding	
25	rs3213623	IVS12‐30C>T	75	25	Non‐coding	
26	**rs34603556**	1337T>C	92.5	7.5	**Met‐>Thr**	**0.00**
27	**rs2301641**	1678A>G	75	25	**Lys‐>Glu**	**1.00**
28	ss836312077^c^	IVS14+50T>C	97.5	2.5	Non‐coding	
29	rs11764760	IVS14‐44T>C	85	15	Non‐coding	
30	rs140377661	IVS15‐28A>T	92.5	7.5	Non‐coding	
31	**ss836312078** ^c^	2166T>G	97.5	2.5	**Asn‐>Lys**	0.92
32	rs5882753	IVS19+17delT	20	80	Non‐coding	
33	rs73085689	IVS20‐30T>C	67.5	32.5	Non‐coding	
34	**rs62453384**	2429G>T	67.5	32.5	**Gly‐>Val**	**0.00**
35	rs62453385	IVS21+95T>C	85	15	Non‐coding	
36	rs4721940	IVS22‐123C>T	67.5	32.5	Non‐coding	
37	rs10254317	2802G>A	37.5	62.5	Ala‐>Ala	
38	**rs6461515**	2908G>A	25	75	**Glu‐>Lys**	0.14
39	rs6461516	IVS24+11C>G	20	80	Non‐coding	
40	rs11380694	IVS24+82_+83insG	40	60	Non‐coding	
41	rs12669866	IVS24‐26T>C	42.5	57.5	Non‐coding	
42	**rs146201784**	3042delT	97.5	2.5	**Leu‐>Stop**	
43	**rs200759253**	3547A>C	97.5	2.5	**Thr‐>Pro**	**0.00**
44	rs189467333	4103G>C	97.5	2.5	Non‐coding	
45	rs182002068	4339G>A	97.5	2.5	Non‐coding	
46	ss836312079^c^	4609A>G	97.5	2.5	Non‐coding	
47	rs12112555	4671G>T	72.5	27.5	Non‐coding	
48	rs150442227	4904G>C	95	5	Non‐coding	
49	rs138210219	4964C>G	95	5	Non‐coding	
50	rs3210441	5014G>A	77.5	22.5	Non‐coding	
51	rs966717	5112T>C	77.5	22.5	Non‐coding	

Abbreviations: Alt. allele, alternative allele; Ref. allele, reference allele; UTR, untranslated region; Var, variantWt, Wild‐type.

^a^ Genetic variants are presented as a nucleotide change from reference allele to alternative allele. Reference allele for a given SNP refers to the nucleotide base on the NCBI reference assembly. For nucleotide change in exon, the name refers to the base position in the ABCB5 cDNA (GenBank acc. no. NM_001163941), with the first base of the ATG start codon set to 1 and the base immediately 5’ is −1. For nucleotide change in intron, the named are expressed as follows: the intron number was followed by a number where n nucleotides upstream of the next exon (−) or downstream (+) of the preceding exon. ins stands for insertion; del stands for deletion.

^b^ Amino acid substitution is classified as ‘Damaging’ if SIFT score is ≤0.05; amino acid substitution is classified as ‘Tolerated’ if SIFT score is >0.05.

^c^ Novel SNPs. NCBI dbSNP has recoded ss1148219560 to rs554561593, ss836312076 to rs869245984, ss836312077 to rs747900970, ss836312078 to rs869152765 and ss836312079 to rs773871763.

### 3.2 ABCB5 genetic variants associated with HCC risk and aggressive tumour features

Specific genetic variants had significant association with important clinicopathological characteristics including tumour size, tumour stage, number of tumour nodules and recurrence‐free survival. Cancer risk was evaluated by comparing the genotypes from blood samples of 300 HCC patients and 300 healthy individuals. The SNPs rs75494098 (IVS2+135 C>T), rs4721940 (IVS22‐123C>T) and rs10254317 (2802G>A) were significantly associated with HCC risk (OR: 0.330, 95% CI: 0.13‐0.84, *P* = .021; OR: 0.363, 95% CI: 0.17‐0.77, *P* = .008; OR: 0.486, 95% CI: 0.27‐0.87, *P* = .014, respectively) (Table 2 and Table S2).

**TABLE 2:** Genetic variants associated with HCC risk

Genotype^a^	HCC (n = 280)	Control (n = 289)	OR (95%CI)	*P* value
rs75494098 (IVS2 + 135C>T)				
CC	274 (97.9%)	271 (93.8%)	1.000 (Ref.)	.021
CT/ TT	6 (2.1%)	18 (6.2%)	0.330 (0.13‐0.84)	

Genotype^a^	HCC (n = 299)	Control (n = 299)	OR (95%CI)	*P* value
rs4721940 (IVS22‐123C > T)				
CC/ CT	289 (96.7%)	273 (91.3%)	1.000 (Ref.)	.008
TT	10 (3.3%)	26 (8.7%)	0.363 (0.17‐0.77)	

Genotype^a^	HCC (n = 296)	Control (n = 299)	OR (95%CI)	*P* value
rs10254317 (2802G > A)				
AA/ AG	277 (93.6%)	262 (87.6%)	1.000 (Ref.)	.014
GG	19 (6.4%)	37 (12.4%)	0.486 (0.27‐0.87)	

Abbreviation: CI, confidence intervals; OR, odds ratios.

^a^ Genotypes of each SNP are arranged in descending order as homozygous dominant, heterozygous and homozygous minor alleles.

Four SNPs, rs73076550 (−90C>T), rs75494098 (IVS2+135C>T), rs76859629 (IVS5+46T>G) and rs12669250 (IVS7+192A>G), are significantly associated with large tumour (OR: 2.352, 95% CI: 1.19‐4.64, *P* = .014; OR: 0.165, 95% CI: 0.03‐0.92, *P* = .040; OR: 0.390, 95% CI: 0.17‐0.91, *P* = .030; OR: 2.316, 95% CI: 1.17‐4.58, *P* = .016, respectively) (Tables S3 and S4). Moreover, 5 SNPs, rs2106562 (−214C>G), rs17143187 (IVS3+1G>C), rs17143212 (392C>T), rs2074000 (784C>A) and rs10254317 (2802G>A), were significantly associated with tumour stage (UICC staging system) (*P* < .05 by chi‐square test or Fisher's exact test as appropriate) (Tables S5 and S6). SNPs rs17143187 (IVS3+1G>C), rs17143212 (392C>T) and rs2074000 (784C>A) had significant association with multiple tumour nodules (OR: 1.936, 95% CI: 1.14‐3.29, *P* = .015; OR: 2.108, 95% CI: 1.16‐3.84, *P* = .015; OR: 1.972, 95%CI: 1.16‐3.34, *P* = .012, respectively) (Tables S7 and S8). Three SNPs in coding region, rs2893006 (1005C>T) in exon 10, rs34603556 (1337T>C) in exon 13 and ss836312078 (rs869152765, 2166T>G) in exon 18 had significant association with disease‐free survival in HCC patient underwent partial hepatectomy (*P* < .001) (Figure S2). Another nine SNPs in the non‐coding region (rs79998607, ss1148219560, rs111872870, rs75494098, rs76859629, rs11769236, rs11772926, ss836312077 and ss836312079) also showed significant association with disease‐free survival outcomes.

**FIGURE 1:**
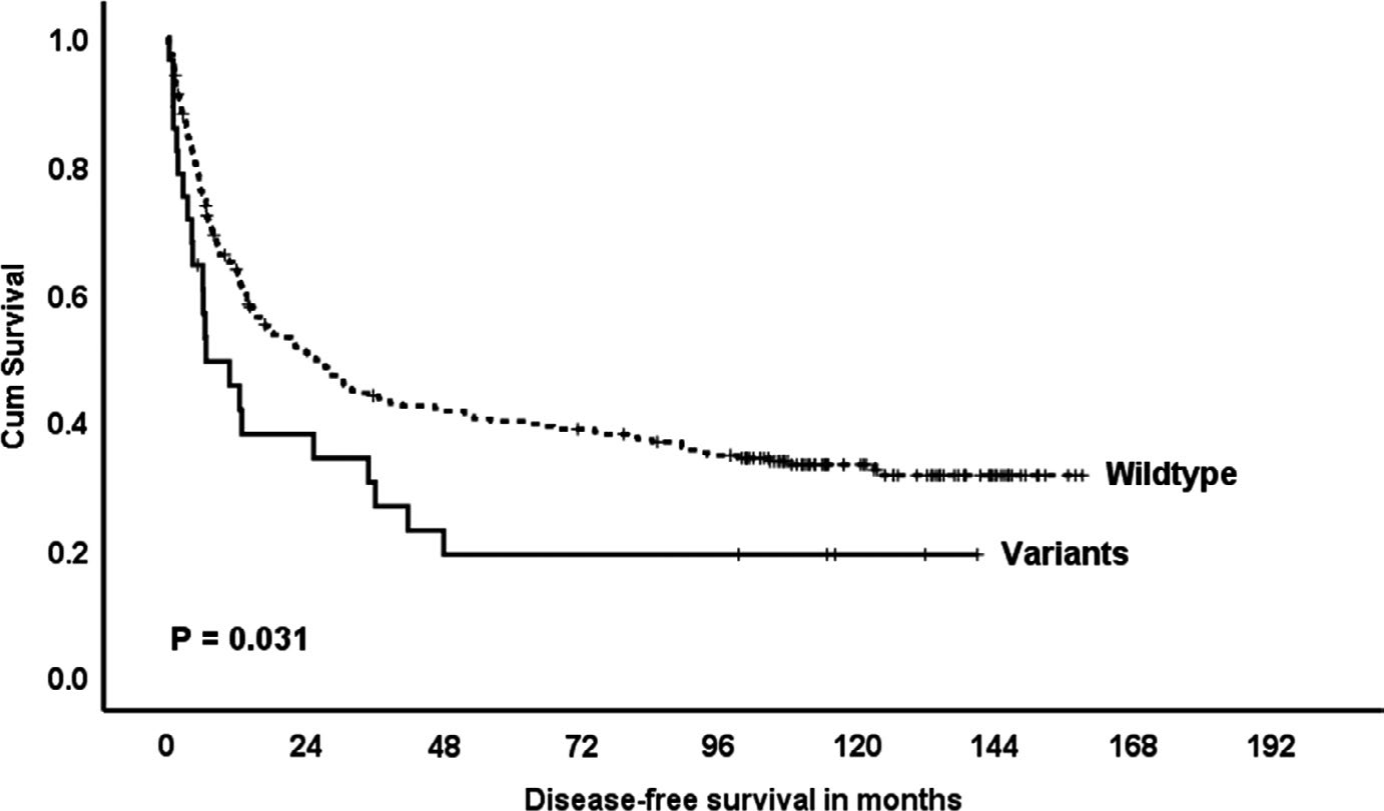
ABCB5 genetic variants and HCC survival outcomes. Three SNPs [rs869152765 (ss836312078), rs11769236 and rs773871763 (ss836312079)] were independent prognostic factors for recurrence‐free survival by Cox regression analysis (OR = 1.622, *P* = 0.033) (N = 295)

**FIGURE 2:**
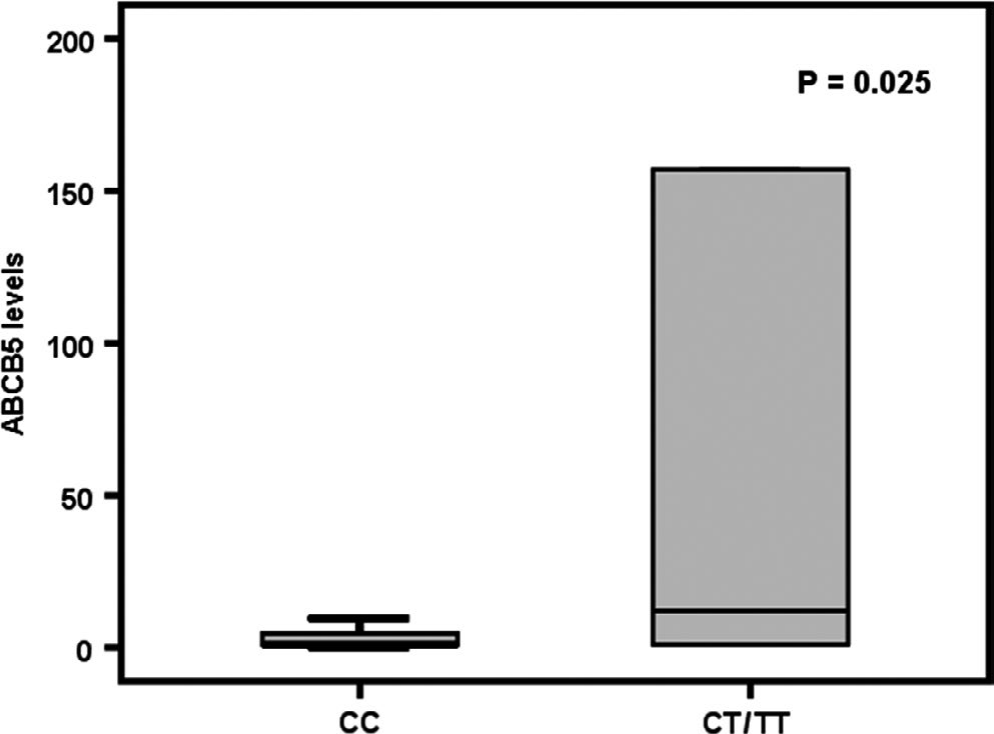
SNP rs17143212 associated with ABCB5 mRNA expression level in HCC

All the 12 SNPs were examined by multivariate Cox regression analysis, and 3 SNPs [rs869152765 (ss836312078), rs11769236 and rs773871763 (ss836312079)] were independent prognostic factors for disease‐free survival (Figure 1). Patients with variant genotypes in these 3 SNPs were grouped as ‘variant’ and patients with only wild‐type genotypes were grouped as ‘wild‐type’ with significant difference (Cox regression, OR = 1.622, *P* = .033; log‐rank test, *P* = .031). Since ABCB5 functionally controlled cancer stemness properties and chemoresistance, the expression levels were analysed with genotypes. SNP rs17143212 (392C>T) was significantly associated with mRNA expression level (*P* = .025, Figure 2). Patients with heterozygous and homozygous variant genotype had higher mRNA expression level in HCC.

### 3.3 ABCB5 expression and mutation status in HCC and other cancer types

**FIGURE 3:**
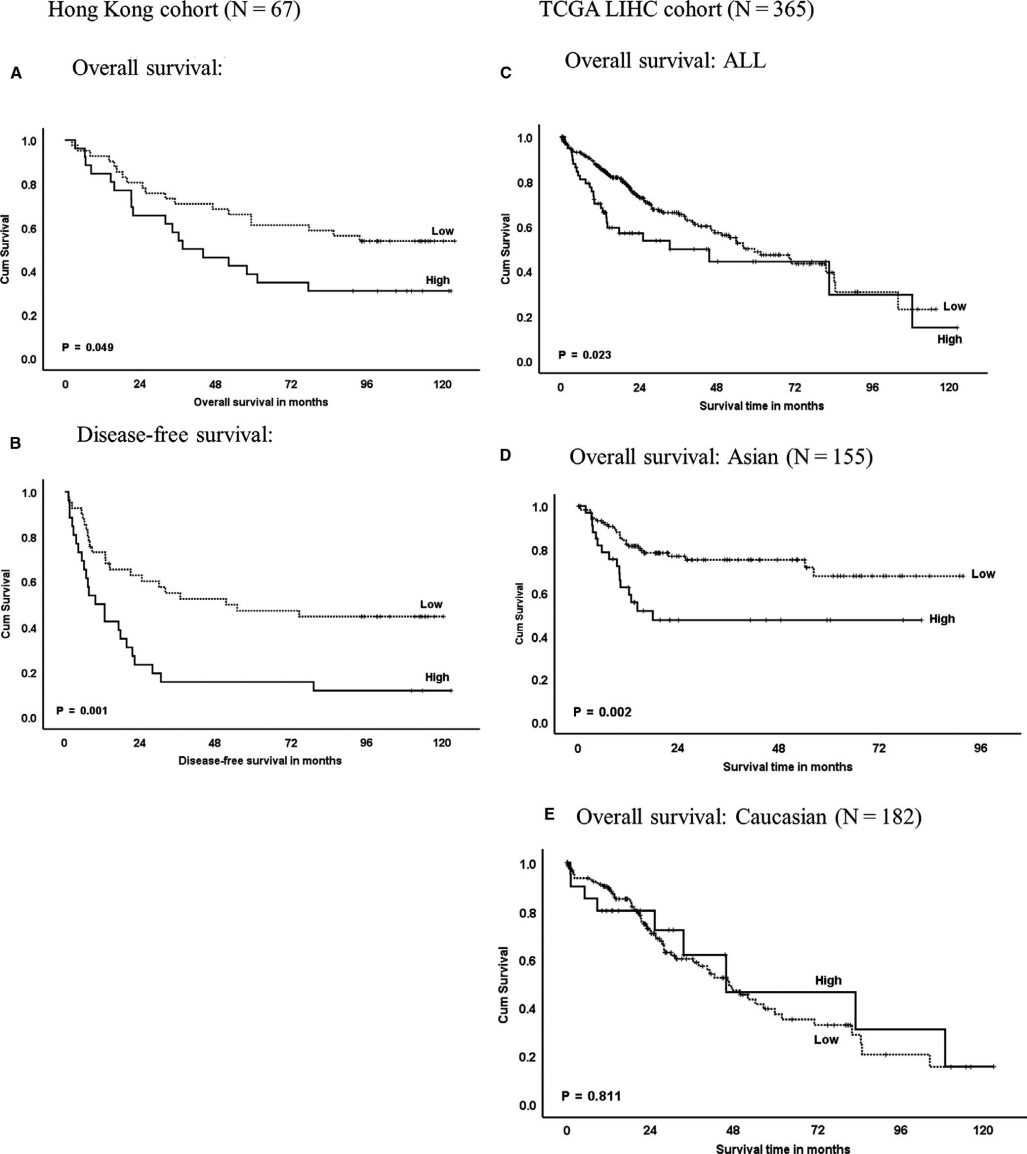
ABCB5 expression associated with poor survival outcomes in Asian HCC patients

Our study revealed that the ABCB5 sequence variation was germline, whereas tumour mutation event was not common. Published HCC genomics cohorts (TCGA, AMC and Inserm) showed corroborating data that ABCB5 mutation rate and copy number variation in HCC was rare event (2.6%, 22/851) (Figure S3), where the ABCB5 expression data were similarly compared to different patient cohorts and cancer types. In Hong Kong Chinese, elevated ABCB5 levels were associated with poor overall survival and disease‐free survival in HCC patients underwent curative surgery (Figure 3A‐B). TCGA HCC dataset revealed similar trend (Figure 3C). Interestingly, when patients were segregated according to ethnic groups, ABCB5 levels demonstrated significant association with survival outcomes only in Asian patients but not in Caucasian patients (Figure 3D‐E). Furthermore, the ABCB5 levels were associated with advanced tumour stage and nodal metastasis (Figure S4). Our data verified the significant association of ABCB5 expression with poor prognosis particularly in Asian HCC patients. Nonetheless, the role of ABCB5 in different populations may not be identical. Further investigations are warranted, in particular the underlying aetiology including alcohol consumption and viral hepatitis.

Additional cancer types were evaluated for the prognostic significance of ABCB5 expression levels. In TCGA datasets, low‐grade glioma (LGG), colon adenocarcinoma (COAD), prostate adenocarcinoma (PRAD) and stomach adenocarcinoma (STAD) all demonstrated that elevated ABCB5 levels were significantly associated with poor overall survival (Figure S5).

### 3.4 Alignment of ABCB5 gene sequences across different species

The human ABCB5 sequence was aligned with sequences from different vertebrates including chimpanzee and mouse (Figure S6). Multiple sequence alignment indicated that the SNPs and INDELs located at the evolutionarily conserved regions. In particular, the ABCB5 sequence variants rs17143212 (392C>T), rs2074000 (784C>A) and rs200759253 (3547A>C) were located at regions evolutionarily conserved among diverse vertebrates indicating the functional importance.

## 4 DISCUSSION

In the present study, we demonstrated that specific ABCB5 genetic variants rs75494098, rs4721940 and rs10254317 were associated with decreased HCC risk. Such finding contrasts with other cancer types. In particular, rs10254317 genotype has been shown to have no significant association with melanoma cancer risk.^22^ Another three SNPs rs10231520, rs17817117 and rs2301641 were reported to associate with decreased melanoma risk,^22^ whereas these SNPs were found in the present HCC study (Table S1) without association with HCC risk. Hence, specific ABCB5 genetic variants were associated with different cancer risks. Remarkably, the majority of all participants in the HCC study were Hong Kong Chinese (99.3%, 298/300), while all were United States Caucasians were investigated in melanoma study.^22^ Therefore, the aetiology including environmental and ethnic factors in such circumstances shall not be underscored.

Among all the SNPs and INDELs identified in this study, 8 SNPs were located in the coding region of ABCB5 gene with amino acid substitutions where 5 of them (rs17143212, rs2074000, rs34603556, rs62453384 and rs200759253) had deleterious amino acid substitutions by SIFT score prediction (Table 1). There was one INDEL (rs146201784) resulting in stop codon and one splicing variant (rs17143187) affecting mRNA splicing, both suggesting detrimental effect on protein function. Among these genetic variants, 3 SNPs rs17143187, rs17143212 and rs2074000 were all associated with aggressive HCC features including advanced tumour stage and multiple nodular tumour. These SNPs conferring unfavourable changes on the transcripts or amino acid alterations shall be the focus for further investigation. Of note, the pilot study examined 20 pairs of HCC and blood samples, and the subsequent validation cohort has investigated 300 HCC and 300 healthy individuals. Low‐frequency variants cannot be identified in the pilot study which could be one of the limitations of the study.

In addition, the potential significance of genetic variants in important functional region of ABCB5 gene including six SNPs identified in the present study (392C>T, 784C>A, 1337 T>C, 1678 A>G, 2429 G>T, 2908 G>A) that were predicted by bioinformatic analysis previously.^17^ For example, 1678 A>G had the lowest subPSEC score (−6.12727) compared to another five SNPs, indicating that 1678 A>G had relatively large impact on ABCB5 function resulting from its dramatic change of amino acid with opposite charged side chain in ATP‐binding domain.^17^ The location of amino acid substitution caused by genetic variants shall be an important factor to determine the degree of deterioration of the protein function. In addition, 392C>T of ABCB5 gene expressed in blood‐brain barrier had been demonstrated to be significantly associated with increased toxicity index in patient cohort treated with haloperidol (*P* = .034).^26^ The 2677 G>A/T leading to amino acid change from alanine to serine or threonine in ABCB1 protein had shown a trend of association with tumour stage in gastric cancer patients (*P* = .0813) and demonstrated significant association with tumour differentiation in colorectal cancer patients (*P* < .05).^26^, ^27^ Moreover, 2677 G>A/T also had significant association with mRNA expression levels in chronic myeloid leukaemia (*P* = .0075), and variant genotypes had higher mRNA expression levels with reference to wild‐type genotype in gastric cancer.^27^, ^28^ Zebrowska et al revealed that the higher the mRNA expression level of ABCB1 gene, the greater anti‐apoptotic effect and thus more advanced stage of cancer was resulted, facilitating tumour progression and aggressiveness.^27^

Elevation of ABCB5 expression has shown to confer cancer stem cell features and drug resistance in melanoma, haematological malignancies and HCC.^7^, ^15^, ^18^, ^19^, ^20^, ^21^ High levels of ABCB5 were associated with poor survival outcomes in HCC (Figure 3) and in diverse cancer types including low‐grade glioma, colon adenocarcinoma, prostate adenocarcinoma and stomach adenocarcinoma as shown in TCGA data sets (Figure S5). The cancer stemness features and chemoresistance would be an obstacle for effective therapy and survival outcome. However, the underlying mechanisms leading to the overexpression of ABCB5 in human cancers remain poorly understood. Our study revealed that somatic mutation in tumour was not common and corroborated with the TCGA data sets. Notably, SNP rs17143212 (392C>T) genetic variants were associated with ABCB5 transcript levels. Moreover, an ABCB1 SNP rs1045642 (3435C>T) had shown to affect mRNA stability^29^ and drug responses.^30^, ^31^ Whether the rs17143212 would alter the ABCB5 mRNA stability and drug sensitivity warranted further analysis.

## 5 CONCLUSIONS

The present study uncovered that specific ABCB5 genotypes were associated with HCC risk, aggressive HCC features, poor recurrence‐free survival and transcript levels. The expression level of ABCB5 mRNA was significantly elevated and associated with poor prognosis of HCC. ABCB5 is a promising HCC biomarker especially among Asian patients. In particular, the germline sequence variation eases the assessment using blood samples, spare the need on tumour biopsy with concern on morbidity and mortality, would facilitate screening and prediction.

## CONFLICT OF INTEREST

The authors confirmed that there are no conflicts of interest.

## AUTHOR CONTRIBUTION

**Idy C. Y. Leung:** Data curation (lead); Formal analysis (lead); Investigation (equal); Methodology (equal); Validation (equal); Writing‐original draft (equal). **Charing C. N. Chong:** Data curation (equal); Formal analysis (equal); Investigation (equal); Writing‐original draft (equal). **Tan‐To Cheung:** Investigation (supporting); Methodology (supporting); Resources (supporting). **Philip Chun Yeung:** Data curation (supporting); Formal analysis (supporting); Validation (supporting); Visualization (supporting). **Kwok Chai Kelvin NG:** Methodology (supporting); Resources (supporting). **Bo San Paul Lai:** Methodology (supporting); Resources (supporting). **LS Chan:** Methodology (supporting); Resources (supporting); Software (supporting). **Anthony Wing‐Hung Chan:** Methodology (supporting); Resources (supporting); Validation (supporting). **Patrick Tang:** Funding acquisition (supporting); Investigation (supporting); Validation (supporting); Visualization (lead); Writing‐review & editing (lead). **Siu Tim Cheung:** Conceptualization (lead); Funding acquisition (lead); Project administration (lead); Resources (lead); Supervision (lead); Writing‐original draft (lead); Writing‐review & editing (lead). 

## Supporting information

Fig S1‐S2

Table S1‐8

Appendix S1‐3

Supplementary Material

## Data Availability

The data that support the findings of this study are available from the corresponding authors upon reasonable request.
