# Supplementary material for: Genetic variation in ABCB5 associates with risk of hepatocellular carcinoma
Source: J Cell Mol Med. 2020 Aug 11;24(18):10705–13. doi: 10.1111/jcmm.15691 (PMC7521249; doi:10.1111/jcmm.15691)

Supplementary Figure 1. Electropherograms of five novel genetic variants identified in ABCB5 gene.

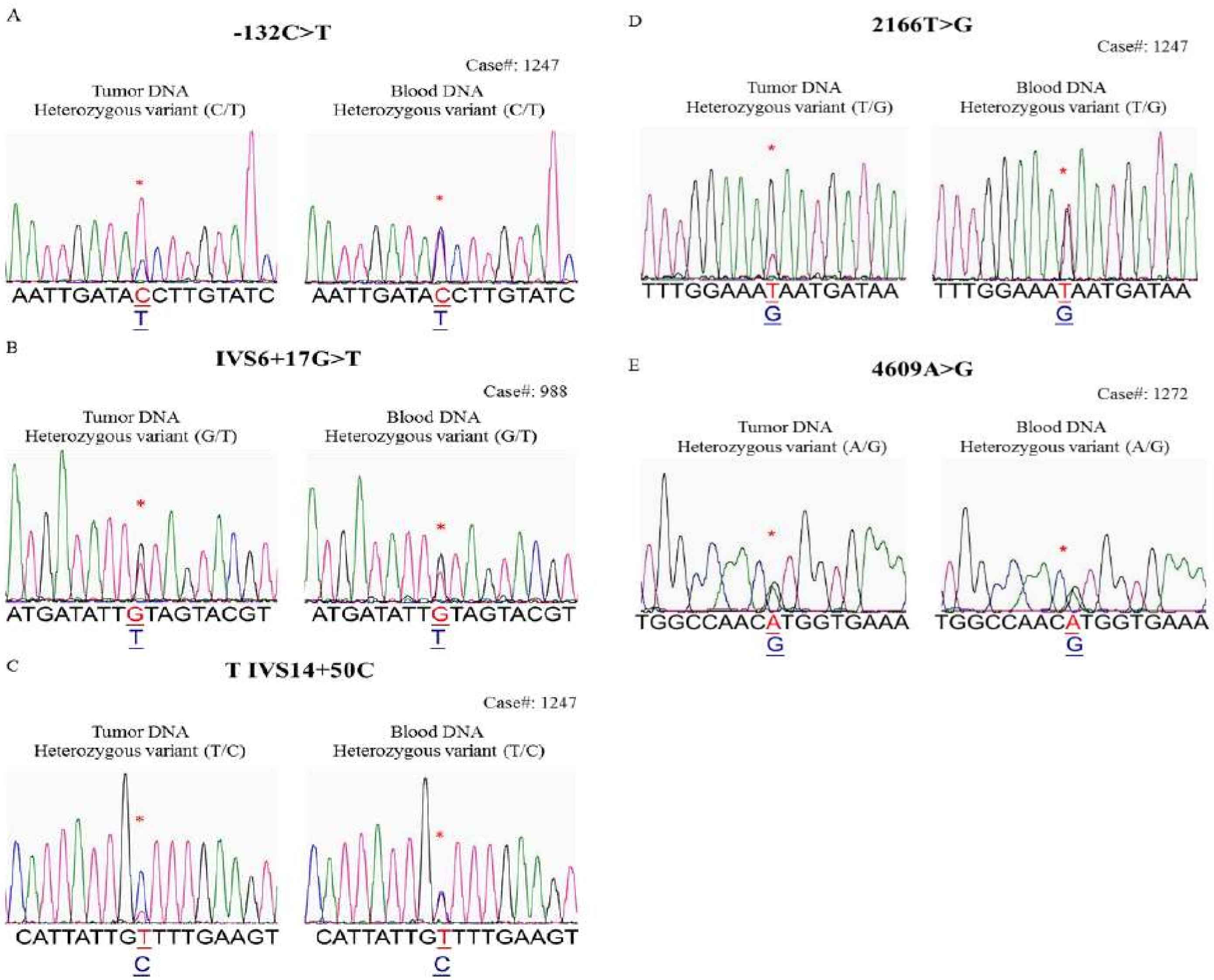

Supplementary Figure 2. Genetic variants in coding region (rs2893006, rs34603556 and ss836312078 (rs869152765)) that had significant association with HCC patient recurrence-free survival. (N=296)

A. SNP rs2893006 (1005C>T) in exon 10

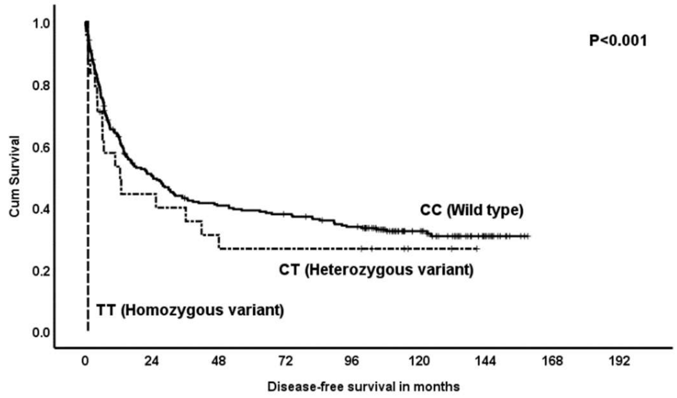

C. SNP ss836312078 (rs869152765, 2166T>G) in exon 18

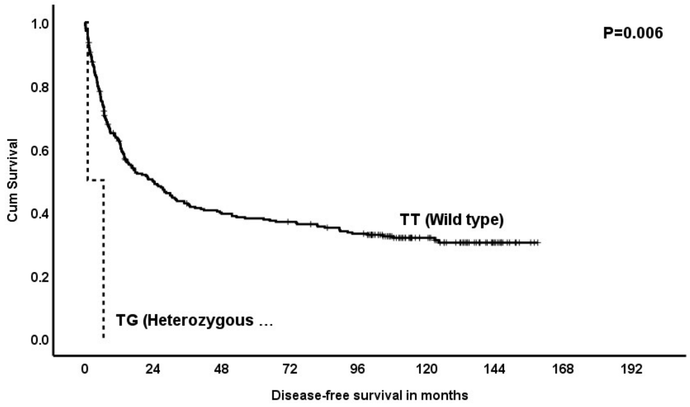

B. SNP rs34603556 (1337T>C) in exon 13

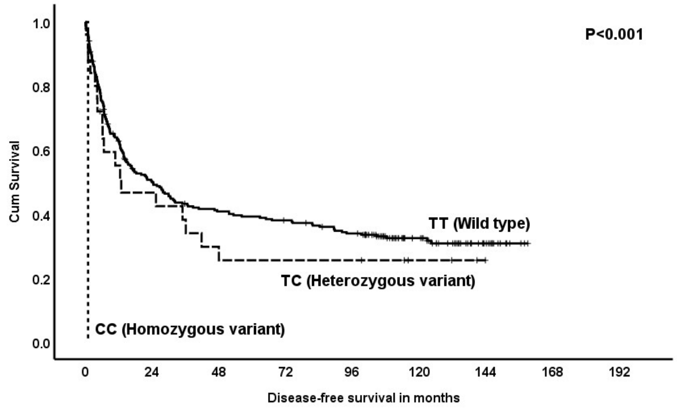

Supplementary Figure 3. HCC genomics datasets (TCGA, AMC and INSERM) showed that ABCB5 mutation rate combined with copy number variation in HCC was rare event (2.6%, 22/851).

A OncoPrint

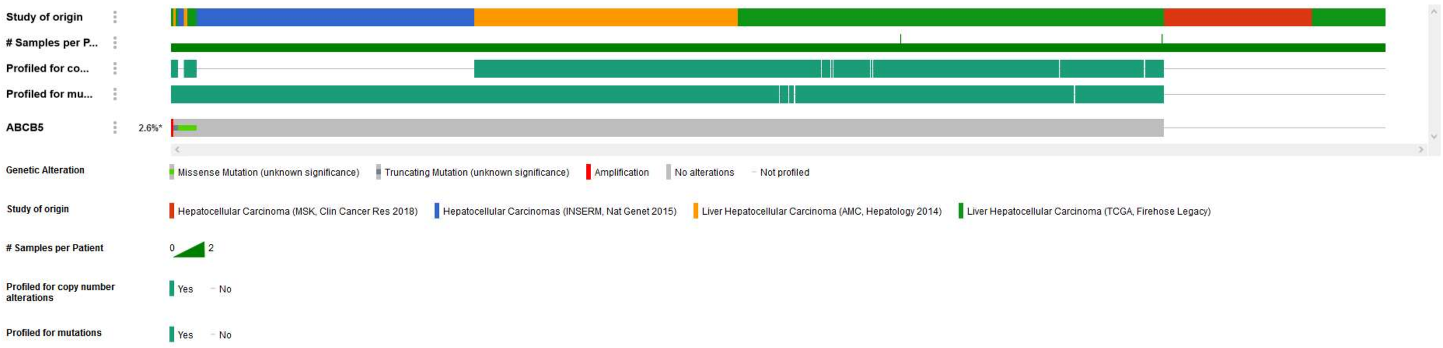

B Cancer Types Summary

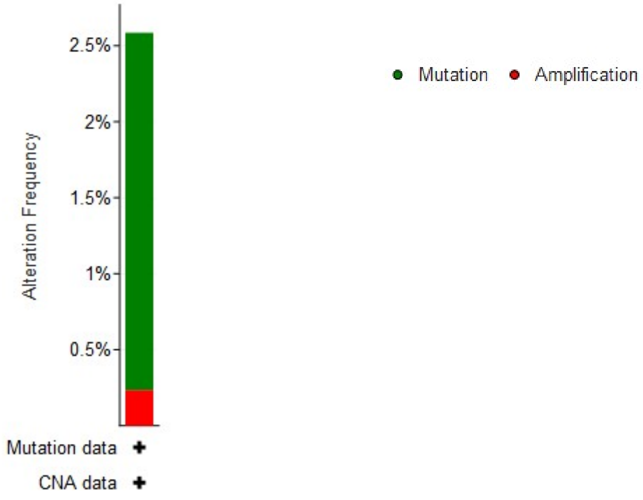

Supplementary Figure 4. ABCB5 transcript levels in TCGA HCC cohorts.

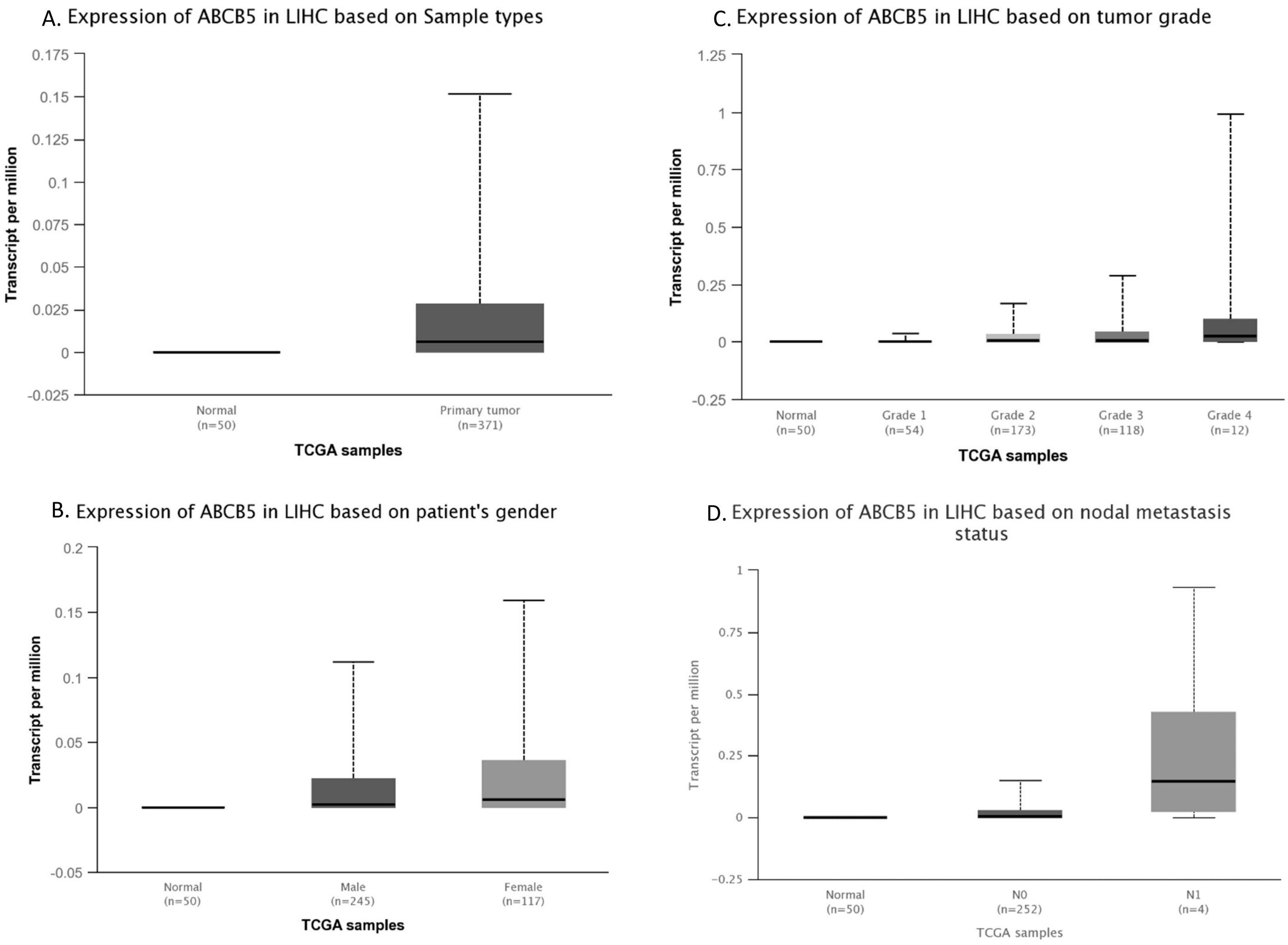

Supplementary Figure 5. Association of ABCB5 expression levels with patient survival in different cancers from TCGA datasets.

A. Low grade glioma (LGG) (N=152)

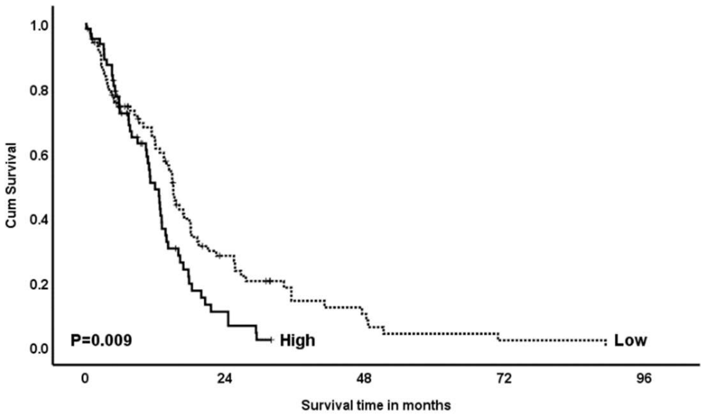

B. Colon adenocarcinoma (COAD) (N=597)

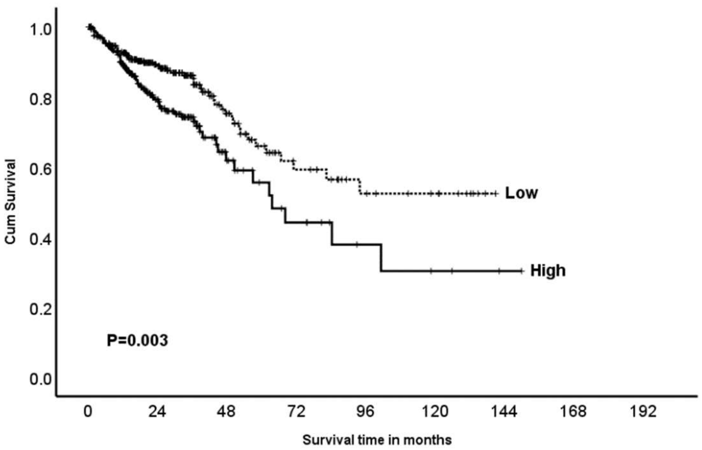

C. Prostate adenocarcinoma (PRAD) (N=494)

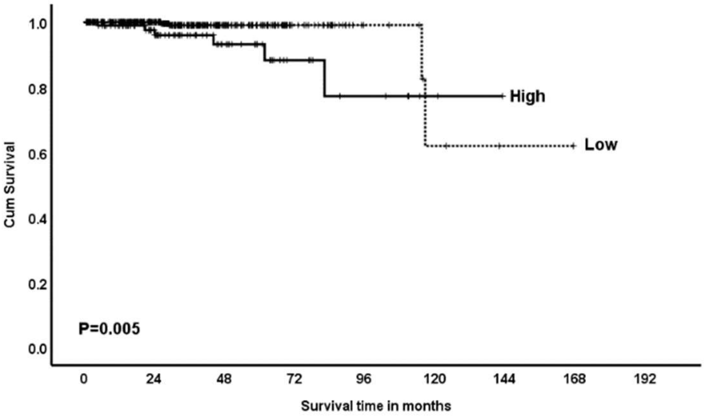

D. Stomach adenocarcinoma (STAD) (N=354)

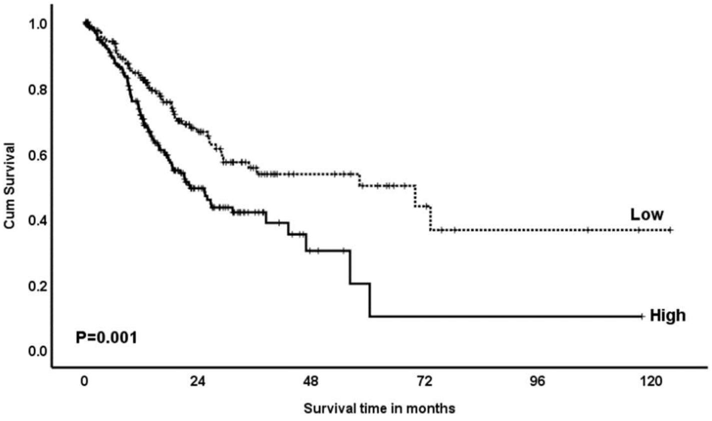

Supplementary Figure 6. Multiple alignment of ABCB5 gene sequence among species.

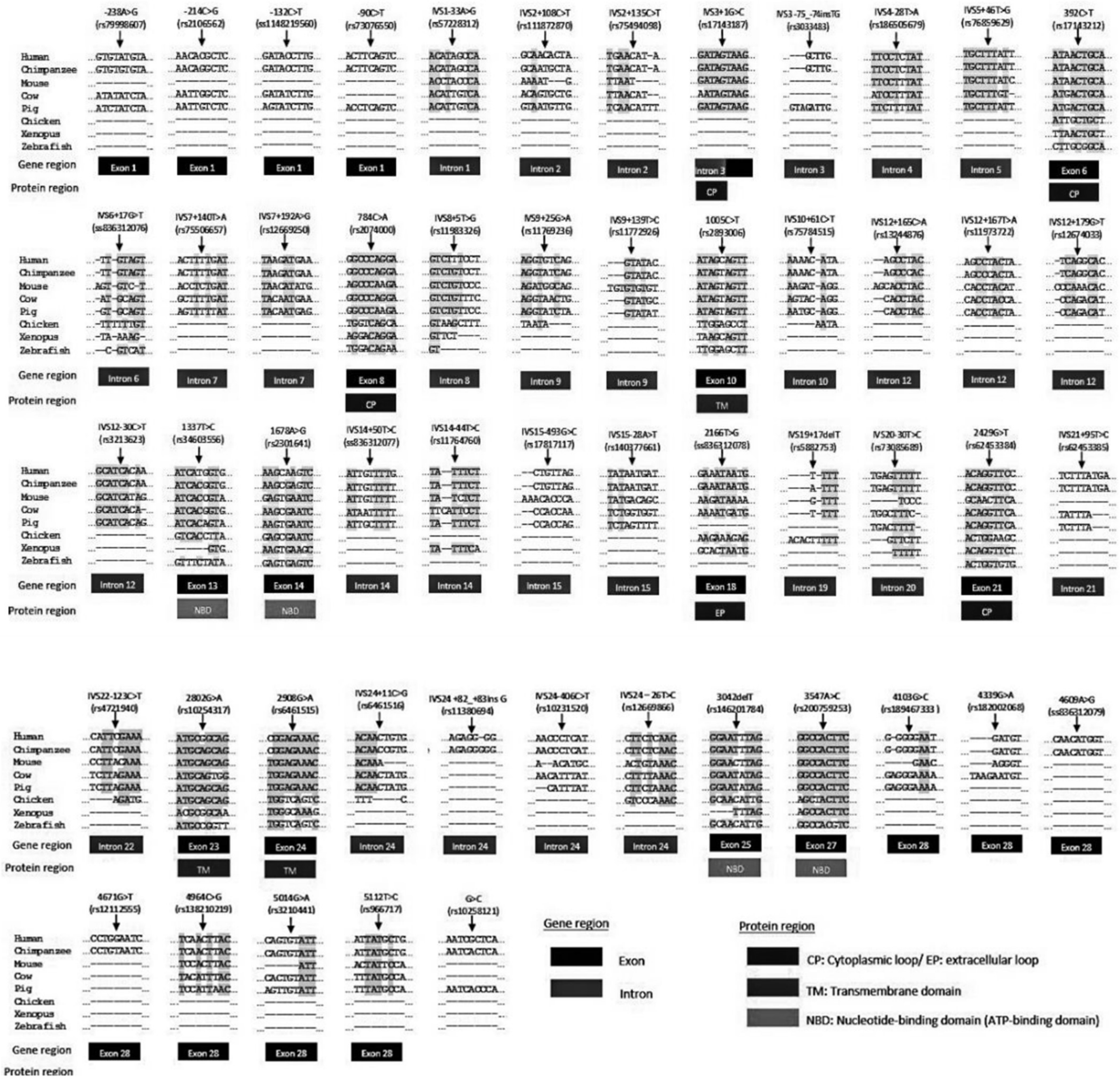

Supplement: Supplementary file 1 — Fig S1‐S2 [file JCMM-24-10705-s001.pdf]
