# Supplementary material for: Genetic variation in ABCB5 associates with risk of hepatocellular carcinoma
Source: J Cell Mol Med. 2020 Aug 11;24(18):10705–13. doi: 10.1111/jcmm.15691 (PMC7521249; doi:10.1111/jcmm.15691)
Supplement: Supplementary file 2 — Table S1‐8 [file JCMM-24-10705-s002.pdf]

Supplementary Table 1. Sequencing platforms used in HCC risk assessment.

Order	dbSNP ID	Nucleotide change	Chromosome Position	Sequencing platform
1	rs79998607	-238A>G	20615621	P
2	rs2106562	-214C>G	20615645	P
3	ss1148219560[#]	-132C>T	20615727	S
4	rs73076550	-90C>T	20615769	S
5	rs57228312	IVS1-33A>G	20623232	S
6	rs111872870	IVS2+108C>T	20623446	S
7	rs75494098	IVS2+135C>T	20623473	S
8	rs17143187	IVS3+1G>C	20626612	S
9	rs3033483	IVS3-75_-74insTG	20628613_20628614	
10	rs186505679	IVS4-28T>A	20632031	
11	rs76859629	IVS5+46T>G	20632159	S
12	rs17143212	392C>T	20643261	S
13	ss836312076[#]	IVS6+17G>T	20643392	S
14	rs75506657	IVS7 +140T>A	20643772	
15	rs12669250	IVS7+192A>G	20643824	S
16	rs2074000	784C>A	20645861	S
17	rs11983326	IVS8+5T>G	20645885	S
18	rs11769236	IVS9+25G>A	20646163	S
19	rs11772926	IVS9+139T>C	20646277	S
20	rs2893006	1005C>T	20647558	S
21	rs75784515	IVS10+61C>T	20647709	
22	rs13244876	IVS12+165C>A	20650312	
23	rs11973722	IVS12+167T>A	20650314	
24	rs12674033	IVS12+179G>T	20650326	
25	rs3213623	IVS12-30C>T	20651390	
26	rs34603556	1337T>C	20651424	S
27	rs2301641	1678A>G	20658647	S
28	ss836312077[#]	IVS14+50T>C	20658726	S
29	rs11764760	IVS14-44T>C	20681461	
30	rs140377661	IVS15-28A>T	20685668	
31	ss836312078[#]	2166T>G	20699836	S
32	rs5882753	IVS19+17delT	20700152	
33	rs73085689	IVS20-30T>C	20722986	
34	rs62453384	2429G>T	20723023	S
35	rs62453385	IVS21+95T>C	20723314	
36	rs4721940	IVS22-123C>T	20728192	P
37	rs10254317	2802G>A	20728390	S
38	rs6461515	2908G>A	20739023	S
39	rs6461516	IVS24+11C>G	20739150	
40	rs11380694	IVS24+82_+ 83insG	20739221_20739222	
41	rs12669866	IVS24-26T>C	20742851	S
42	rs146201784	3042delT	20742894	S
43	rs200759253	3547A>C	20753477	S

44	rs189467333	4103G>C	20755953	S
45	rs182002068	4339G>A	20756189	S
46	ss836312079[#]	4609A>G	20756459	P
47	rs12112555	4671G>T	20756521	S
48	rs150442227	4904G>C	20756754	S
49	rs138210219	4964C>G	20756814	S
50	rs3210441	5014G>A	20756864	S
51	rs966717	5112T>C	20756962	S
52	rs17817117	IVS15-493G>C	20685203	S
53	rs10231520	IVS24-406C>T	20742471	S
54	rs10258121	G>A	20759290	S

Abbreviations: S, SNP genotyping; P, Pyrosequencing

Note: Genetic variants 1-51 were identified in pilot study based on Hong Kong HCC cohort. Genetic variants 52-53 were reported to be significantly associated with reduced melanoma risks, and genetic variant 54 was one of the 44 tagging SNPs of ABCB5 in the same melanoma study (Lin et al., 2013). Chromosome position of SNPs or INDELs referred to the position at chromosome 7 of NCBI human reference genome (GRCh38.p2).

[#] Novel SNPs. NCBI dbSNP has recoded ss1148219560 to rs554561593, ss836312076 to rs869245984, ss836312077 to rs747900970, ss836312078 to rs869152765, and ss836312079 to rs773871763.

Supplementary Table 2. Genetic variants associated with HCC risk.

rs75494098 (IVS2+135C>T)

Genotype	HCC (n=280)	Control (n=289)	OR (95%CI)	P value
CC	274 (97.9%)	271 (93.8%)	1.000 (Ref.)	0.042
CT	5 (1.8%)	18 (6.2%)	0.275 (0.10-0.75)	0.012
TT	1 (0.4%)	0 (0%)	1.60 x 10^9 (0)	1.000

rs4721940 (IVS22-123C>T)

Genotype	HCC (n=299)	Control (n=299)	OR (95%CI)	P value
CC	163 (54.5%)	163 (54.5%)	1.000 (Ref.)	0.022
CT	126 (42.1%)	110 (36.8%)	1.145 (0.82-1.60)	0.428
TT	10 (3.3%)	26 (8.7%)	0.385 (0.18-0.82)	0.014

rs10254317 (2802G>A)

Genotype	HCC (n=296)	Control (n=299)	OR (95%CI)	P value
AA	134 (45.3%)	136 (45.5%)	1.000 (Ref.)	0.036
GA	143 (48.3%)	126 (42.1%)	1.152 (0.82-1.62)	0.412
GG	19 (6.4%)	37 (12.4%)	0.521 (0.29-0.95)	0.034

Abbreviation: OR: odds ratios; CI: confidence intervals

Supplementary Table 3. Genetic variants associated with tumor size (n=295).

Genetic variants	Genotype*	Tumor size ≤3cm	Tumor size >3cm	P^
rs73076550 (-90C>T)	CC	66	152	0.013
	CT	8	57	
	TT	4	8	
rs75494098 (IVS2+135C>T)	CC	67	203	0.016#
	CT	4	1	
	TT	0	1	
rs76859629 (IVS5+46T>G)	TT	67	203	0.047#
	GT	11	12	
	GG	0	1	
rs12669250 (IVS7+192A>G)	AA	66	152	0.020
	AG	8	55	
	GG	4	9	

^ P value of χ^2 test, except # P value of Fisher's exact test as appropriate.

Supplementary Table 4. Regression analysis for genetic variants associated with tumor size (n=295).

Genetic variants	Genotype*	Tumor size ≤3cm	Tumor size >3cm	OR (95%CI)	P value
rs73076550 (-90C>T)	CC	66	152	1.000 (Ref.)	
	CT / TT	12	65	2.352 (1.19-4.64)	0.014
rs75494098 (IVS2+135C>T)	CC	67	203	1.000 (Ref.)	
	CT / TT	4	2	0.165 (0.03-0.92)	0.040
rs76859629 (IVS5+46T>G)	TT	67	203	1.000 (Ref.)	
	GT / GG	11	13	0.390 (0.17-0.91)	0.030
rs12669250 (IVS7+192A>G)	AA	66	152	1.000 (Ref.)	
	AG / GG	12	64	2.316 (1.17-4.58)	0.016

Supplementary Table 5. Genetic variants associated with tumor stage (n=295).

Genetic variants	Genotype*	Tumor stage Early Stage	Late Stage	P^
rs2106562 (-214C>G)	GG	165	68	0.004#
	GC	28	28	
	CC	3	0	
rs17143187 (IVS3+1G>C)	GG	42	40	0.001
	GC	102	39	
	CC	52	17	
rs17143212 (392C>T)	CC	165	72	0.042#
	CT	28	24	
	TT	3	0	
rs2074000 (784C>A)	CC	42	42	<0.001
	CA	101	37	
	AA	53	17	
rs10254317 (2802G>A)	AA	77	52	0.017
	AG	100	42	
	GG	17	2	

^ P value of χ^2 test, except # P value of Fisher's exact test as appropriate.

Supplementary Table 6. Regression analysis for genetic variants associated with tumor stage (n=295).

Genetic variants	Genotype*	Tumor stage		OR (95%CI)	P value
		Early Stage	Late Stage		
rs2106562 (-214C>G)	GG	165	68	1.000 (Ref.)	
	CC / CG	31	28	2.192 (1.22-3.93)	0.008
rs17143187 (IVS3+1G>C)	GG	42	40	2.619 (1.54-4.45)	<0.001
	GC / CC	154	56	1.000 (Ref.)	
rs17143212 (392C>T)	CC	165	72	1.000 (Ref.)	
	CT / TT	31	24	1.774 (0.97-3.23)	0.061
rs2074000 (784C>A)	CC	42	42	2.852 (1.68-4.84)	<0.001
	CA / AA	155	54	1.000 (Ref.)	
rs10254317 (2802G>A)	AA / AG	177	94	1.000 (Ref.)	
	GG	17	2	0.222 (0.50-0.98)	0.047

Supplementary Table 7. Genetic variants associated with tumor nodules (n=295).

Genetic variants	Genotype*	Tumor nodules		P^
		One	Multiple	
rs17143187 (IVS3+1G>C)	GG	48	35	0.045
	GC	105	38	
	CC	49	20	
rs17143212 (392C>T)	CC	172	68	0.035[#]
	CT	28	24	
	TT	2	1	
rs2074000 (784C>A)	CC	49	36	0.039
	CA	102	38	
	AA	51	19	

^ P value of χ^2 test, except [#] P value of Fisher's exact test as appropriate.

Supplementary Table 8. Regression analysis for genetic variants associated with tumor nodules (n=295).

		Number of tumor nodules			
Genetic variants	Genotype*	One	Multiple	OR (95%CI)	P value
rs17143187 (IVS3+1G>C)	GG	48	35	1.936 (1.14-3.29)	0.015
	GC /CC	154	58	1.000 (Ref.)	
rs17143212 (392C>T)	CC	172	68	1.000 (Ref.)	
	CT / TT	30	25	2.108 (1.16-3.84)	0.015
rs2074000 (784C>A)	CC	49	36	1.972 (1.16-3.34)	0.012
	CA / AA	153	57	1.000 (Ref.)	
