# Supplementary material for: Genetic variation in ABCB5 associates with risk of hepatocellular carcinoma
Source: J Cell Mol Med. 2020 Aug 11;24(18):10705–13. doi: 10.1111/jcmm.15691 (PMC7521249; doi:10.1111/jcmm.15691)
Supplement: Supplementary file 3 — Appendix S1‐3 [file JCMM-24-10705-s003.pdf]

Appendix 1 PCR primers and sequencing primers for DNA sequencing

PCR no.	Amplified exon	PCR primers	Sequencing primer	Amplicon Length (bp)	Annealing temperature (℃)	Extension Time (s)
1	Exon 1	Forward: 5’-CTAAAAATTGGTTCAGTGCCT<br>Reverse: 5’-AACATGGGATCTTTGGTTAGA	Reverse: 5’-ATACACACACATTTTTGTTTCCA	1145	59	68
2	Exon 2	Forward: 5’-TCCCTCAAACTGCTGGCTAATA<br>Reverse: 5’-TGCAGGTGCCTTTCTTCTGTATA	Forward: 5’-CCTAGAATGAAGCAAGGAAGTTATA	807	61	45
3	Exon3	Forward: 5’-CAACCAGTGACAACTGTCTATCA<br>Reverse: 5’-TGAAACGCTCCTTAAACATGA	Forward: 5’-TTCAGTGTTGGTATAAATTTGCT	740	55	45
4	Exon 4	Forward: 5’-CTGCTTTGTTTTTTAATGCCT<br>Reverse: 5’-GCAATATGGCAGAATAAATGACT	Reverse: 5’-GCAATATGGCAGAATAAATGACT	529	55	32
5	Exon 5	Forward: 5’-AAGCCGCATGAGTACTTCTT<br>Reverse: 5’-GCATATGACCTATACACCTTTCA	Forward: 5’-TGAGATGATAATAAACTGCGTGT	466	55	30
6	Exon6, 7	Forward: 5’-TTTTACCAGGAAACCTAGATCA<br>Reverse: 5’-TATCCATGCTACCACTTGAAGT	Forward: 5’-TCTAGCATAAATTTCCTCAATCA (Exon 6)<br>Forward: 5’-TGTGACATCGGTGAACTTAACA (Exon 7)	906	59	57
7	Exon8, 9	Forward: 5’-TCACCAATAGTGATATTTTCCCT<br>Reverse: 5’-ACAAAACTCAGTGTATTTCCCA	Forward: 5’-TTTCTCTCTGAAATCTTACCTCTT (Exon 8)<br>Forward: 5’-ATCCGAACAGTCATAGCCTTTA (Exon 9)	862	59	57
8	Exon 10, 11	Forward: 5’-TTGGCTAAGATCAAGTGTAATCT<br>Reverse: 5’-CACGGAGCTGAAAATGCT	Forward: 5’-CCATTCTTTCTTACCTAATTCCT (Exon 10)<br>Forward: 5’-TTGAAACCTTCATGTCTAACTCA (Exon 11)	948	59	57
9	Exon 12	Forward: 5’-TTGTGAAAATATCTGGCAAGA<br>Reverse: 5’-CAACATTCCCTCCCTTCTAA	Forward: 5’-CTGAAATTATTCAGGCTATTACTACA	806	59	57
10	Exon 13	Forward: 5’-CAGAGGTGTGAGATGTTGAAAT<br>Reverse: 5’-CATACAGGAGTGTCCAATTTTT	Forward: 5’-TTCTAAAATATGCTACTTCTCAGCT	933	59	57
11	Exon 14	Forward: 5’-CAGTGGTTCTTTTAAAGATTTCA<br>Reverse: 5’-CACTTGGAATGGAGGTATAGACT	Forward: 5’-GGCTGAAGTACTGATTAAGCTGA	778	59	46
12	Exon 15	Forward: 5’-CTCTGCCCAAGGATAAGATACT<br>Reverse: 5’-CTCCATTTCTTTACTGAGGAACT	Forward: 5’-CCCAAAGTGCTGGAATTACA	921	55	57
13	Exon 16	Forward: 5’-GCCCAGCCTCACATATAAGTA<br>Reverse: 5’-ATGGTTTGCTTCATAGGAAAA	Forward: 5’-ATCAGCTCTCTATCAAGCCTGA	821	55	57
14	Exon 17	Forward: 5’-TAATGAAACTTTTACCTTCCACA<br>Reverse: 5’-TGCAACTATACCCACTAATTGAA	Forward: 5’-TCATCGTTAATAATACGGGCA	902	55	57
15	Exon 18, 19	Forward: 5’-GCATTGAGCCAAGACTGAGAT<br>Reverse: 5’-TTTAACGTCACACTTTTGGACTT	Reverse: 5’-CCCTGAAAAGCAAACAAACA (Exon 18)<br>Forward: 5’-CCTGAGCATGATTACTTTTTGTT (Exon 19)	692	57	42
16	Exon 20	Forward: 5’-TCATCGCCTTACTTTTGAATTA<br>Reverse: 5’-CTTGGTCTCTTCTTTAGTCCTCTA	Forward: 5’-GCAACAGTCATTCAATCCATAGT	692	59	46
17	Exon 21	Forward: 5’-GGTATTCCACTAAACTAAAATCCA<br>Reverse: 5’-AGAAACCTGAAGTCTACAAAGGT	Forward: 5’-CTCAAAGTATAAATTCAAGGCAT	1054	54	68
18	Exon 22	Forward: 5’-TGGGGTTAAACTTAGATCAGTAGA<br>Reverse: 5’-TGCCCTCAATTCCTCTTCTA	Forward: 5’-TATTTTCTGGGAGGAAAGGAA	522	59	35

19	Exon 23	Forward: 5’-AATGGAGACTTAGAAAATCCTTCT<br>Reverse: 5’-GTAAATAAGTACATGACCCCCTT	Reverse: 5’-ACACCCAGCTAATTTTTGTATTT	859	59	52
20	Exon 24	Forward: 5’-TTCTCTGTCAAATCTCCAAGCA<br>Reverse: 5’-ACATTCTCATAGCATCAGGCATAT	Forward: 5’-GGTGTACAGAGCAAGGAATGAT	979	61	59
21	Exon 25	Forward: 5’-TGGTTTTTAGAAACTCAGTGAAGT<br>Reverse: 5’-TTAGTTAAGGGTTGACACAGAGAT	Forward: 5’-GCAGTCAACAGCTCTACAGAGAT	590	59	35
22	Exon 26	Forward: 5’-GCTGTTTGCTTCTATACAACGTA<br>Reverse: 5’-GCATCTGACTTCATGTTTTACAT	Forward:5’-ACTTTGAAATAGCTTGAATTCCT	536	59	35
23	Exon 27	Forward: 5’-AAGAGCCAAAGTGAAGAAACTT<br>Reverse: 5’-TCTGACCCTGCAGTGTAGATTA	Forward: 5’-GCCCAAGAATAGATTTCAACA	957	59	57
24	Exon 28	Forward: 5’-TTTTACAAGTAGGGCAGTTCCT<br>Reverse: 5’-CTTCTTTGCATTACACGAACAT	Forward: 5’-AAGCAAATAAAGCAAAGAAGGT	383	59	25
25	Exon 28	Forward: 5’-GCAAAGAAGGTTATTAGACTACCT<br>Reverse: 5’-CCCAGAAATTTTACATCGTAAT	Forward: 5’-AATTCAGAACGCAGATTTGATA	828	59	50
26	Exon 28	Forward: 5’-CAATACCTAGAATCATGCTACTCA<br>Reverse: 5’-TTCACCCACAATAAAATTAGCT	Forward: 5’-AACTATTCTAGCACATTTGCTTGT<br>Reverse: 5’-GTTCAAATGATTCTCCTGCCT<br>Reverse: 5’-AGTTCTGGGATTACAGGCGT	960	59	57
27	Exon 28	Forward: 5’-ACGATGTAAAATTTCTGGGAGGA<br>Reverse: 5’-TGTGTCTAAGCAATGCATCTGATT	Forward: 5’-ACGCCTGTAATCCCAGAACT	847	59	57
28	Exon28	Forward: 5’-GTTGAGCAGGGAATAGAAAGGA<br>Reverse: 5’-GGTATGGGACAGATGTTGATAGGT	Forward: 5’-GAGCTAATTTTATTGTGGGTGA	1103	58	66

Appendix 2 PCR primers and extension primers for SNP genotyping

PCR no.	dbSNP ID	PCR primers	Extension primer	Amplicon Length (bp)
1	ss1148219560	Forward: 5'-ACGTTGGATGCCAAAATCCACAAGCCAGAC<br>Reverse: 5'-ACGTTGGATGAGTCTTCTGAAGAGGATAGG	Forward: 5'-GGAAAATTCTGGCAAAAAATTGATA	114
2	rs73076550	Forward: 5'-ACGTTGGATGGAAAGCTTTATCCTATCCTC<br>Forward: 5'-ACGTTGGATGCATCCAGTCAGTTGAAGTCC	Reverse: 5'-AGTCCTTAAGAGAAAAGACT	88
3	rs57228312	Forward: 5'-ACGTTGGATGGTGGGATGTAAAGAATGCTG<br>Reverse: 5'-ACGTTGGATGTTTACAATTTACTTCTTCTG	Forward: 5'-AAACTAAGTGATAAAAGTCACAT	118
4	rs111872870	Forward: 5'-ACGTTGGATGTAACTCATCCTTTCCACACC<br>Reverse: 5'-ACGTTGGATGTTCAAGTTCAGCAAAATGC	Forward: 5'-ATTTTTTCCCAAAAATAGCAA	107
5	rs75494098	Forward: 5'-ACGTTGGATGCTATTTGCATTTTGCTGAA<br>Reverse: 5'-ACGTTGGATGCCTATTGCAGTTCCTAAAATC	Forward: 5'-TATTGGCATTTTGCTGAACTTGAA	96
6	rs17143187	Forward: 5'-ACGTTGGATGCTGAGAAAGGAAGCAGTTGG<br>Reverse: 5'-ACGTTGGATGATGCACTACTGACAAGTCTC	Forward: 5'-AGCAGTTGGATCTATTGAGATA	116
7	rs76859629	Forward: 5'-ACGTTGGATGGTCATACAAAATTTCAGTTGC<br>Reverse: 5'-ACGTTGGATGCTGTAAGTCCAAATGAAACG	Reverse: 5'-TGCTTTTTTCTTGTAAGCTTTAAATA	112
8	rs17143212	Forward: 5'-ACGTTGGATGTCGAATCCTCTTGGTCTGTC<br>Reverse: 5'-ACGTTGGATGGAATAGGTGTTGCTGCCTTG	Reverse: 5'-AGTCTGTCGTGCTGCA	106
9	ss836312076	Forward: 5'-ACGTTGGATGGTGACATCGGTGAACTTAAC<br>Reverse: 5'-ACGTTGGATGGTTAGGTCATTTACATGTCAC	Forward: 5'-TACAGAGTAAGAGGATGATATT	117
10	rs12669250	Forward: 5'-ACGTTGGATGGTTTTCAATGTTAGTAAGC<br>Reverse: 5'-ACGTTGGATGTTTGTTCACCATTAATCCCC	Reverse: 5'-GTTAGTAAGCTATTAAAAACATTTCA	103
11	rs2074000	Forward: 5'-ACGTTGGATGCTGGGGAAAATAAAACCAAGC<br>Reverse:5'-ACGTTGGATGCAATCCGAACAGTCATAGCC	Reverse: 5'-CCTTTGAAGTTCTTTCTCCT	118
12	rs11983326	Forward: 5'-ACGTTGGATGCAATCCGAACAGTCATAGCC<br>Reverse: 5'-ACGTTGGATGCTGGGGAAAATAAAACCAAGC	Forward: 5'-GAGAAAGAACTTCAAAGGTCT	118
13	rs11769236	Forward: 5'-ACGTTGGATGTGGATATACCATCGGGACTG<br>Reverse: 5'-ACGTTGGATGTTTGGCTAAACTAGGTAAGG	Forward: 5'-GGAGGTCTTGTTTGAGAACAAGGT	112
14	rs11772926	Forward: 5'-ACGTTGGATGGCGAGGACACTAAAACATGC<br>Reverse: 5'-ACGTTGGATGACAAATATTTGTTTCCCTC	Reverse: 5'-ATTCATAACTATAATTTAAAGGTGTAT	117
15	rs2893006	Forward: 5'-ACGTTGGATGGTTTCAAAGTGAGGGACTGC<br>Reverse: 5'-ACGTTGGATGGTTTGTTCCTGTAGGTTTTC	Reverse: 5'-TGCTCCAATGCAATAACT	96
16	rs34603556	Forward: 5'-ACGTTGGATGACCACTCCAATATGGTCTCG<br>Reverse: 5'-ACGTTGGATGGGTTCATTCTTTGGATTGGC	Reverse: 5'-TGTCATTCTCATCCACC	110
17	rs2301641	Forward: 5'-ACGTTGGATGTTCTCCAGTGCAGCTTGAAC<br>Reverse: 5'-ACGTTGGATGTCGTGCCTTAGTTCGAAACC	Reverse: 5'-GCTTGAACAGCTGACT	119
18	ss836312077	Forward: 5'-ACGTTGGATGGGCTCTCAACTTGAGTAATC<br>Reverse: 5'-ACGTTGGATGGTGAGCAGAAACGTTTCTTA	Reverse: 5'-TCTATACTTTCTTGTACTTCAAA	118

19	ss836312078	Forward: 5'-ACGTTGGATGTTCCCCCAAACACCTGATAA<br>Reverse: 5'-ACGTTGGATGCTGCATCATGCTTTAATGTG	Forward: 5'-CCCCTTTTCAGATGTTTGGAAA	101
20	rs62453384	Forward: 5'-ACGTTGGATGGAAAGTCCCATGTTAGTTGC<br>Reverse: 5'-ACGTTGGATGTTTCCCCCAAAATATGTCTG	Reverse: 5'-ACGCCAATCCTGGAA	103
21	rs10254317	Forward: 5'-ACGTTGGATGGATTATTGGAAGCTGTTATGC<br>Reverse: 5'-ACGTTGGATGGCTTGAATTAAATAGGCTCC	Forward: 5'-GGCCTTTATATATTTTGCCTATGC	110
22	rs6461515	Forward: 5'-ACGTTGGATGACTGCAATTGCATATGGAGC<br>Reverse: 5'-ACGTTGGATGCAAGGCAAACAGATGCGCAG	Forward: 5'-GTGTAGCTATGGCCATCGGA	119
23	rs12669866	Forward: 5'-ACGTTGGATGTCCTGTGAAAGGAAGTTGAC<br>Reverse: 5'-ACGTTGGATGGAACTTGGCCAGTATGCTAC	Reverse: 5'-AAGGAAGTTGACAGAGTTG	99
24	rs146201784	Forward: 5'-ACGTTGGATGAACTTCCTTTCACAGGACAC<br>Reverse: 5'-ACGTTGGATGAAAACATCTGGGCGACATGG	Forward: 5'-ACACATGTGAAGGGAATT	103
25	rs200759253	Forward: 5'-ACGTTGGATGACTAGCTATTGCAAGGGCTC<br>Reverse: 5'-ACGTTGGATGTCTCACTGTCATTAGCGAGG	Reverse: 5'-TAGCGAGGGCTGAAG	106
26	rs189467333	Forward: 5'-ACGTTGGATGTATTTCCCATCAACTTCTGC<br>Reverse: 5'-ACGTTGGATGCTGCCTTTGCTAAAACCTTC	Reverse: 5'-AACATGGTTAATTGGATAATATTC	112
27	rs182002068	Forward: 5'-ACGTTGGATGTAGCTACCTAATCCTCCCAG<br>Reverse: 5'-ACGTTGGATGATAAGTATTCACTATTTCTC	Reverse: 5'-CTACTCCTCCCAGAAATTTTACAT	119
28	rs12112555	Forward: 5'-ACGTTGGATGCTGCCTCCTAGGTTCAAATG<br>Reverse: 5'-ACGTTGGATGATTAGCCAATCTTGGTGGCG	Reverse: 5'-GCCAAGTAGCTGGGATT	104
29	rs150442227	Forward: 5'-ACGTTGGATGCTCTATAATAAAATAATTTCC<br>Reverse: 5'-ACGTTGGATGCCAATATTGTTATTTACACAG	Reverse: 5'-CACAGAACTTTAAAATCATTCA	114
30	rs138210219	Forward: 5'-ACGTTGGATGAAATCACCAGGTGACATTAC<br>Reverse: 5'-ACGTTGGATGCTGTGTAAATAACAATATTGG	Reverse: 5'-AGGTGACATTACATATTTAAGTAA	95
31	rs3210441	Forward: 5'-ACGTTGGATGTGTCACCTGGTGATTTTATC<br>Reverse: 5'-ACGTTGGATGAGGGAGGCTAGCTAAATGTG	Forward: 5'-GGGTGATTTTATCTTTATTCTTCAGT	90
32	rs966717	Forward: 5'-ACGTTGGATGCTCAAGTCCCCAATCTTATT<br>Reverse: 5'-ACGTTGGATGCTACCAATAATTGAAATCTTG	Forward: 5'-GCATTTTAAAGAAAGCAGC	119
33	rs17817117	Forward: 5'-ACGTTGGATGGCACCTGGTCTTTAATGTTA<br>Reverse: 5'-ACGTTGGATGACCCATTCTGAACTGTGAAC	Forward: 5'-TGAGGAGTAGGCCCT	119
34	rs10231520	Forward: 5'-ACGTTGGATGAAACATGGCCCAATTTAGAG<br>Reverse: 5'-ACGTTGGATGGCTTAATTCAGAAGAGCTGG	Forward: 5'-GCCCAATTTAGAGAAATAACC	93
35	rs10258121	Forward: 5'-ACGTTGGATGACTTCAGCAGATTTAAATG<br>Reverse: 5'-ACGTTGGATGAGCTGTACACCTAAGCATGG	Forward: 5'-TTTAAATGTAAGTTTTACATGTTAAATC	113

Appendix 3 PCR primers and sequencing primers for pyrosequencing

PCR no.	dbSNP ID	PCR primers	Sequencing primer	Amplicon Length (bp)
1	rs4721940	Forward: 5’-CCATCCTTTCACGGGCTTT<br>Reverse: 5’-GGCATGAATTATAGCAAGGTCAA (biotinlyated)	Forward: 5’-TCACTTTCTCTTTCATCATT	261
2	rs2106562	Forward: 5’-GTCTTGCCAGGTATATTAGGTTCC (biotinlyated)<br>Reverse: 5’-TTTTGGAAGTGCCAGTCACC	Reverse: 5’-CACCACAATTACATGAGC	146
3	ss1148219560	Forward: 5’-GCAGTATAAGTCACAGGCCTACC (biotinlyated)<br>Reverse: 5’-CCTCTGCCTCCTAGGTTCAAA	Reverse: 5’-AGAGACGGGGTTTCAC	284
4	rs79998607	Forward: 5’-AGAAATAGAATCAATAGGCCGTGT (biotinlyated)<br>Reverse: 5’-TTCTAGTCTGGCTTGTGGATTTT	Reverse: 5’-GTTTTCTCTTTCTCTCTCTA	136
