# Supplementary material for: Genetic variation in ABCB5 associates with risk of hepatocellular carcinoma
Source: J Cell Mol Med. 2020 Aug 11;24(18):10705–13. doi: 10.1111/jcmm.15691 (PMC7521249; doi:10.1111/jcmm.15691)
Supplement: Supplementary file 4 — Supplementary Material [file JCMM-24-10705-s004.docx]

Supplementary Figure 1. Electropherograms of five novel genetic variants identified in ABCB5 gene.


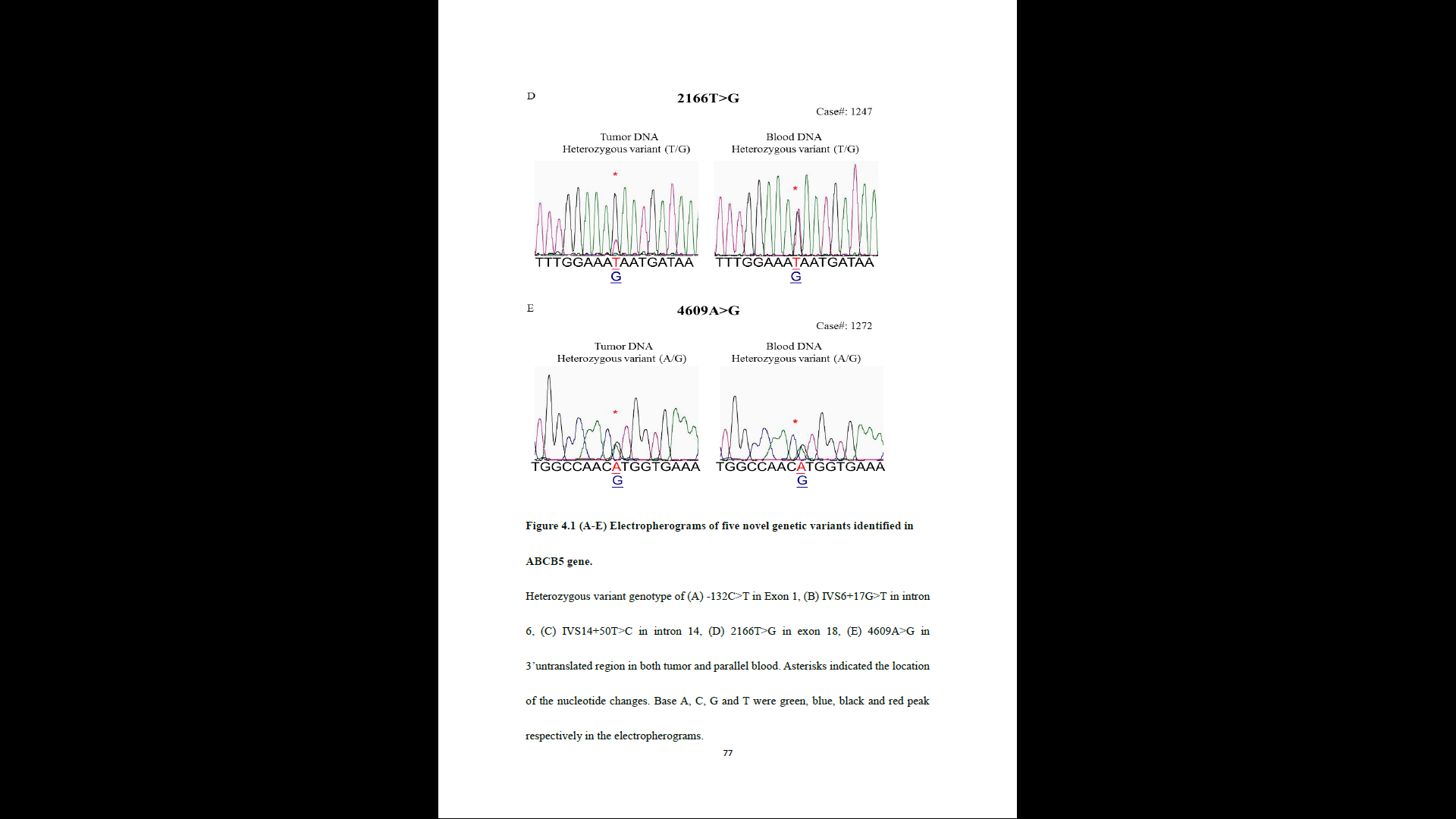


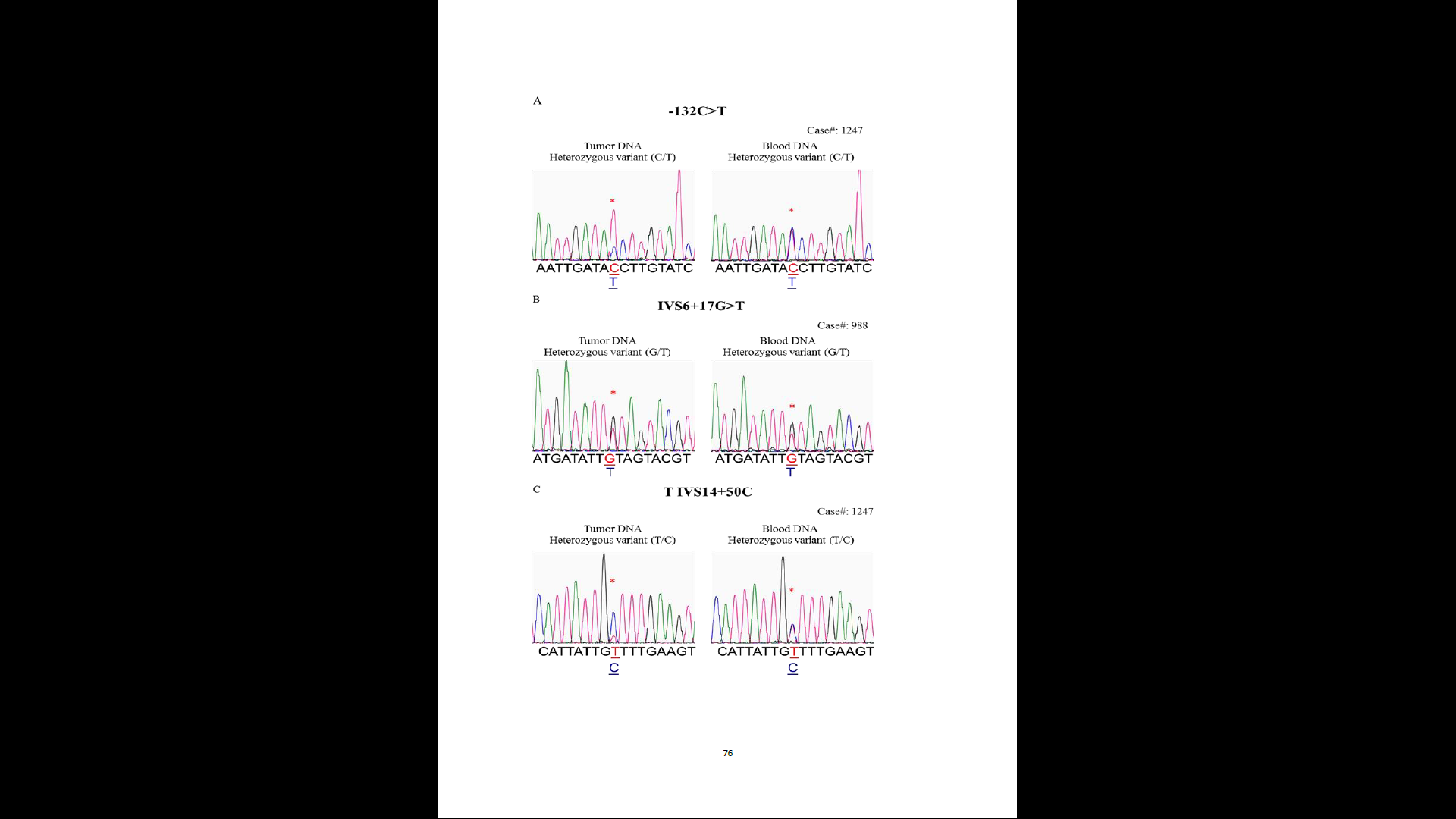


Supplementary Figure 2. Genetic variants in coding region (rs2893006, rs34603556 and ss836312078 (rs869152765)) that had significant association with HCC patient recurrence-free survival. (N=296)

| A. SNP rs2893006 (1005C>T) in exon 10  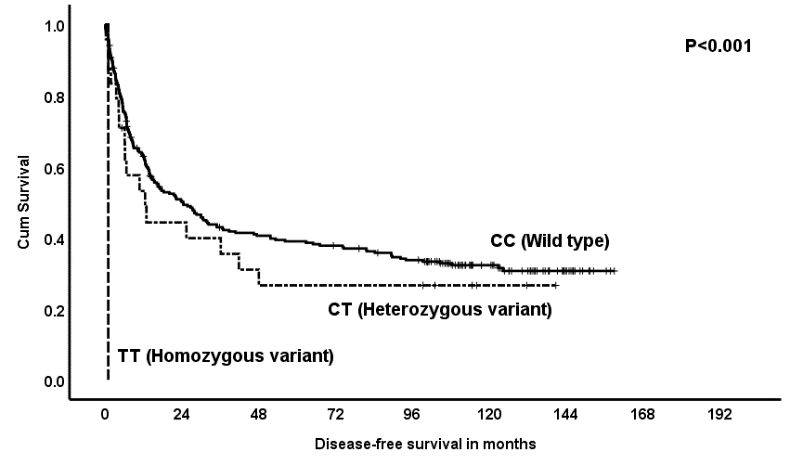 | C. SNP ss836312078 (rs869152765, 2166T>G) in exon 18  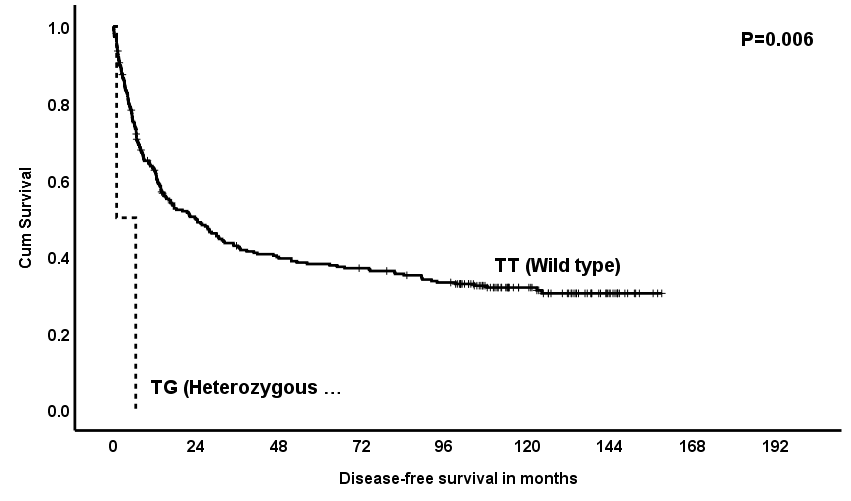 |
| --- | --- |
| B. SNP rs34603556 (1337T>C) in exon 13  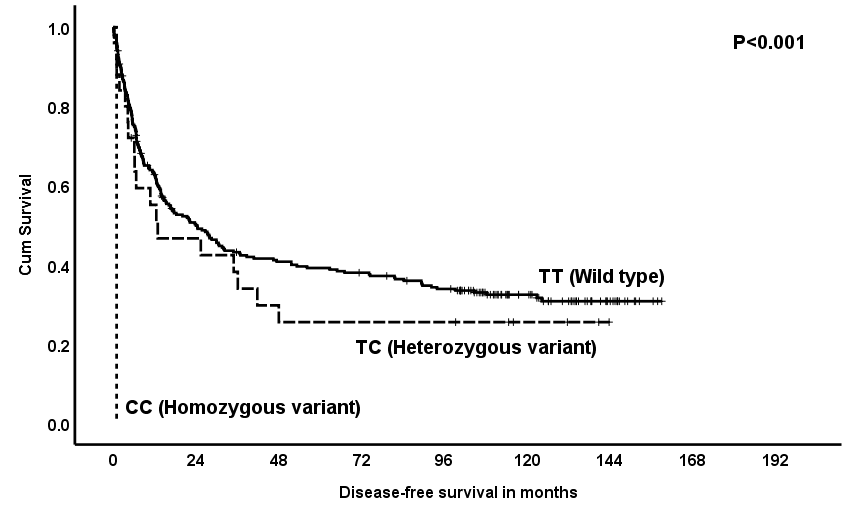 |  |

Supplementary Figure 3. HCC genomics datasets (TCGA, AMC and INSERM) showed that ABCB5 mutation rate combined with copy number variation in HCC was rare event (2.6%, 22/851).

A OncoPrint


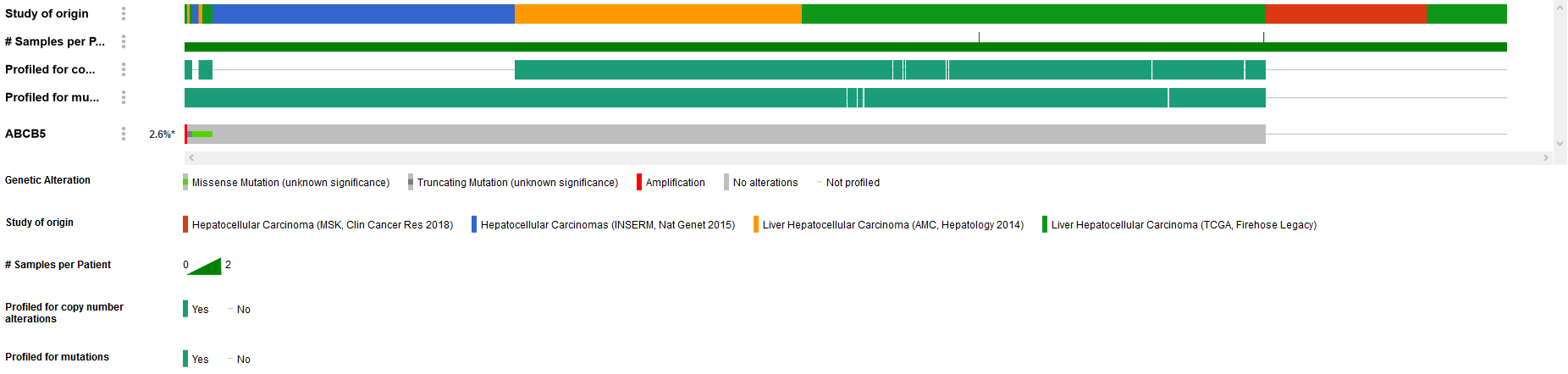


B Cancer Types Summary


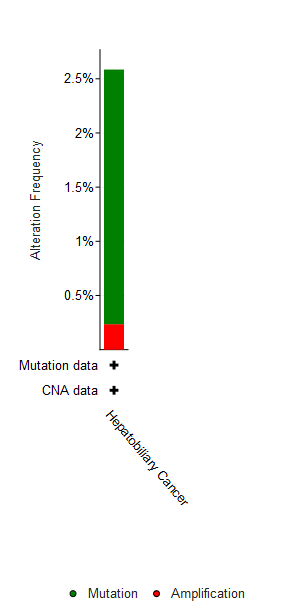


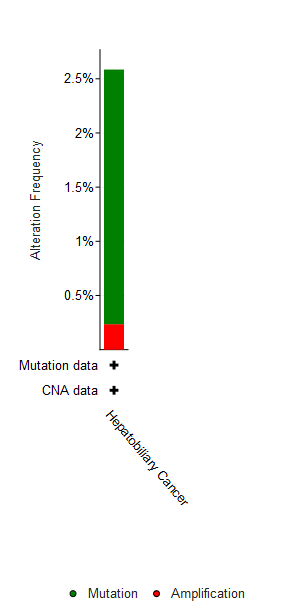


Supplementary Figure 4. ABCB5 transcript levels in TCGA HCC cohorts.

C.

A.


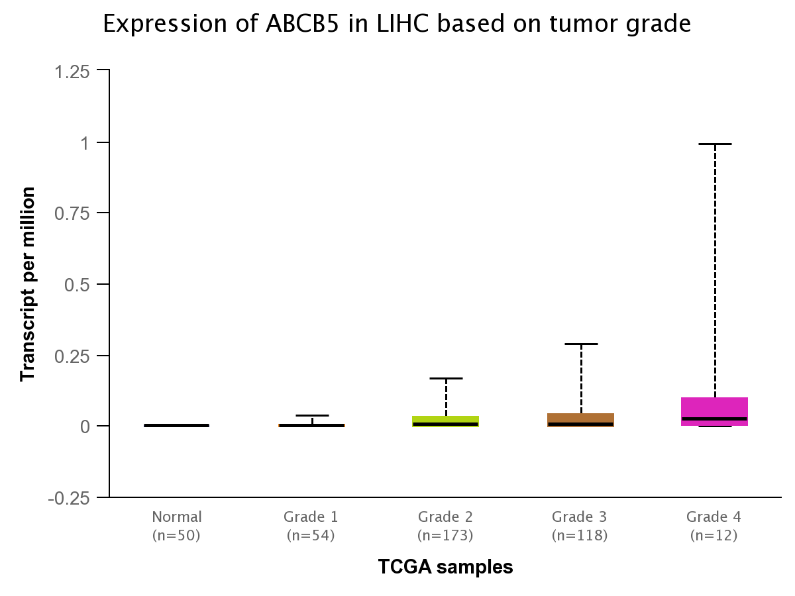

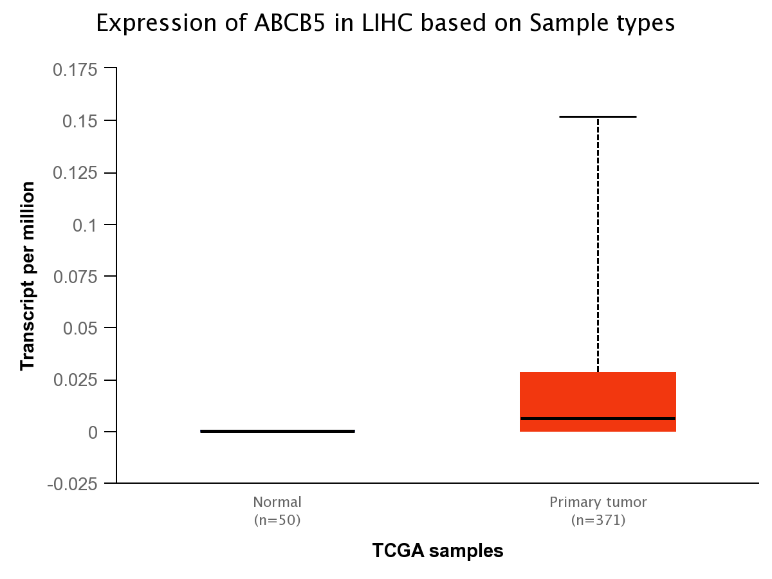


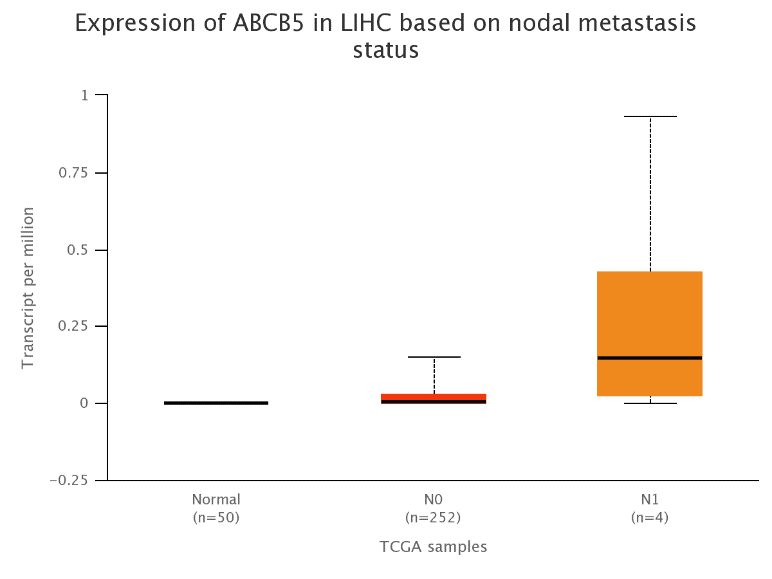

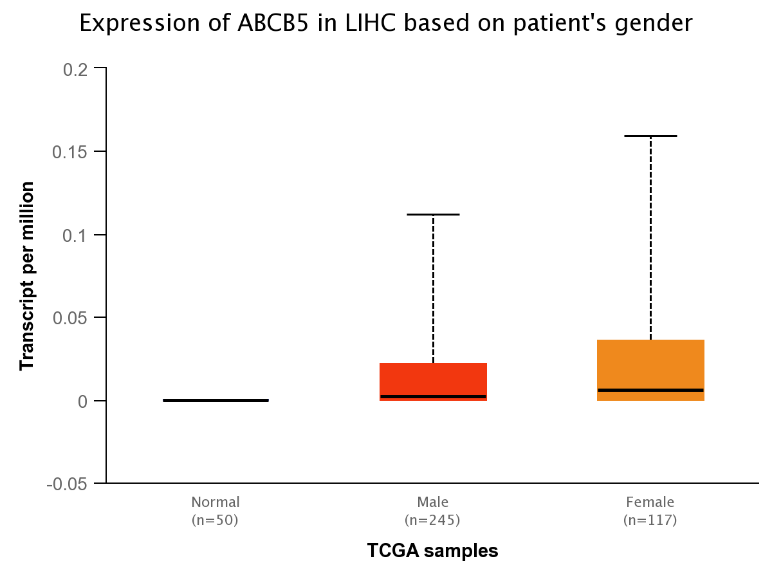


D.

B.

Supplementary Figure 5. Association of ABCB5 expression levels with patient survival in different cancers from TCGA datasets.


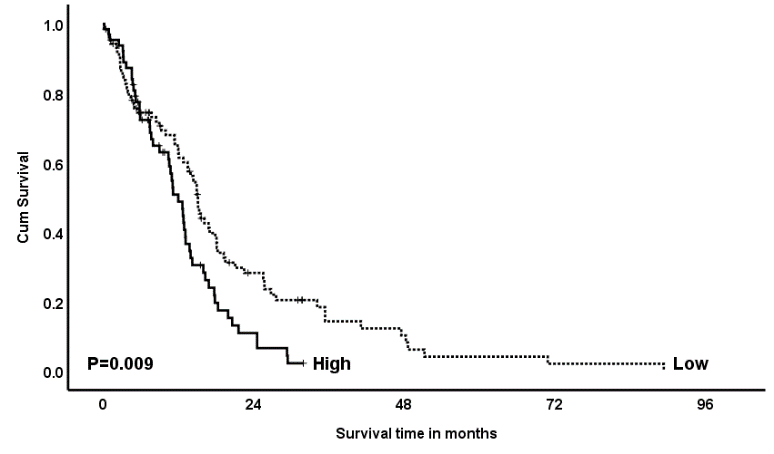


| A. Low grade glioma (LGG) (N=152)  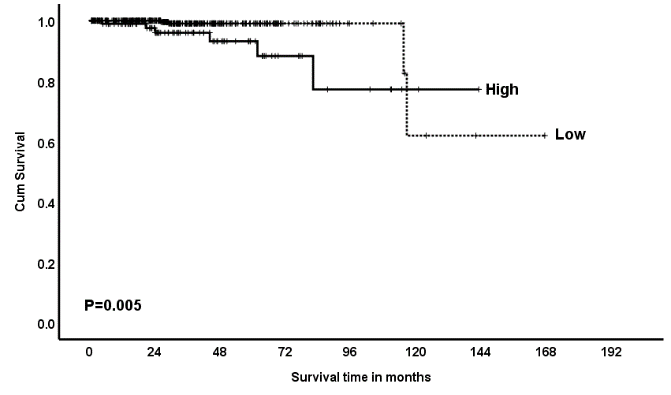 | C. Prostate adenocarcinoma (PRAD) (N=494) |
| --- | --- |
| B. Colon adenocarcinoma (COAD) (N=597)  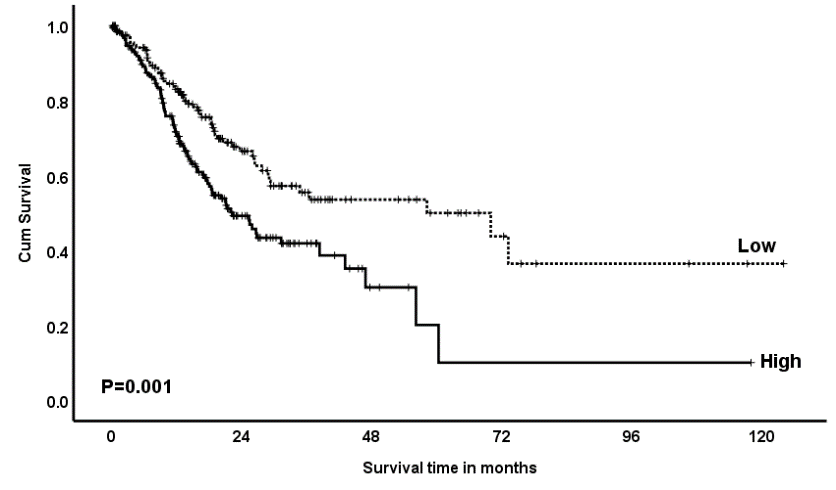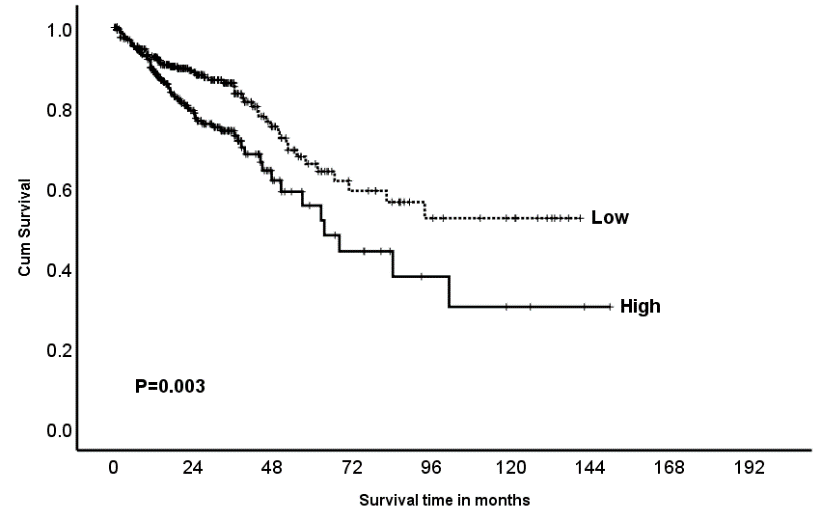 | D. Stomach adenocarcinoma (STAD) (N=354) |


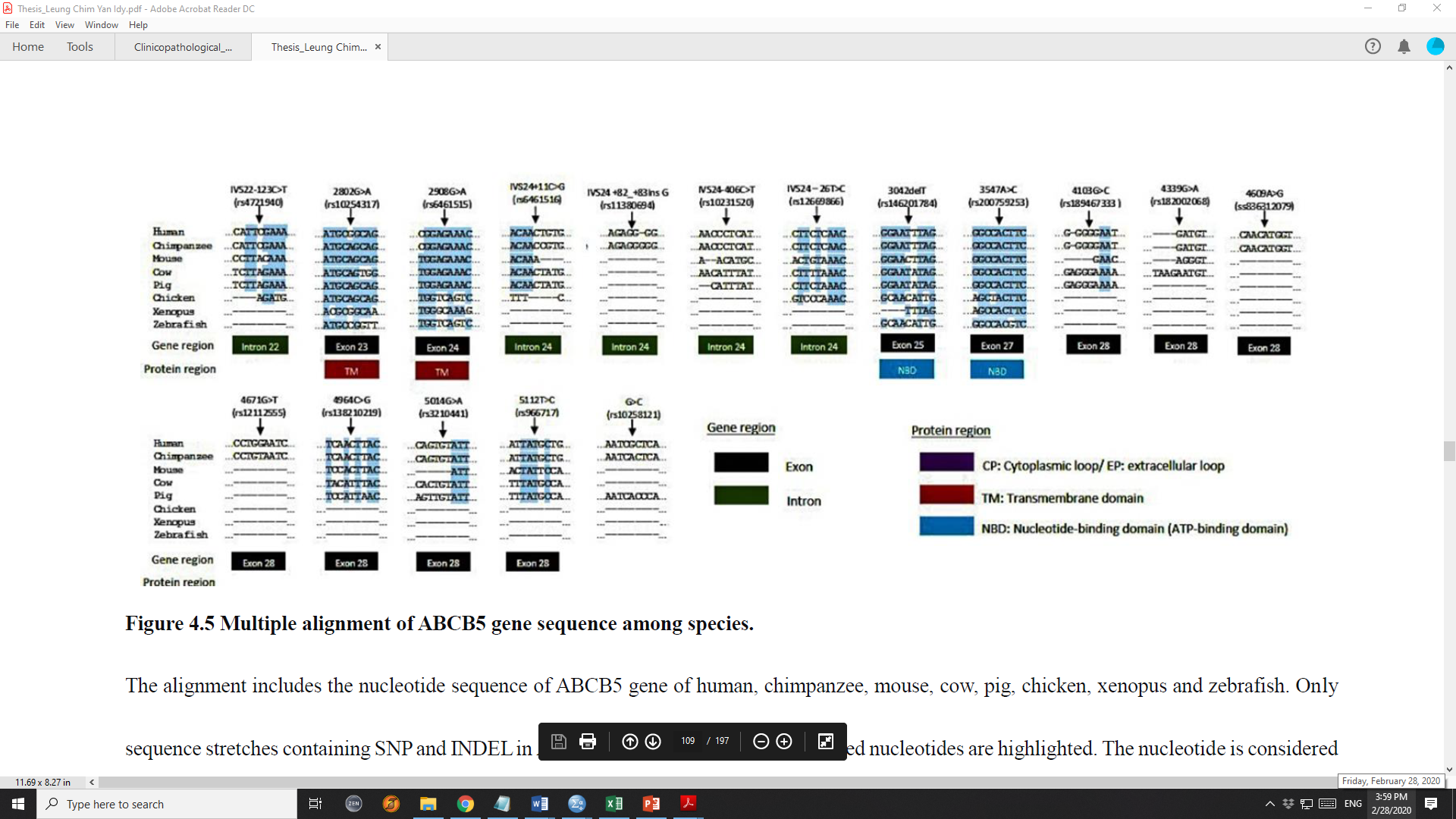

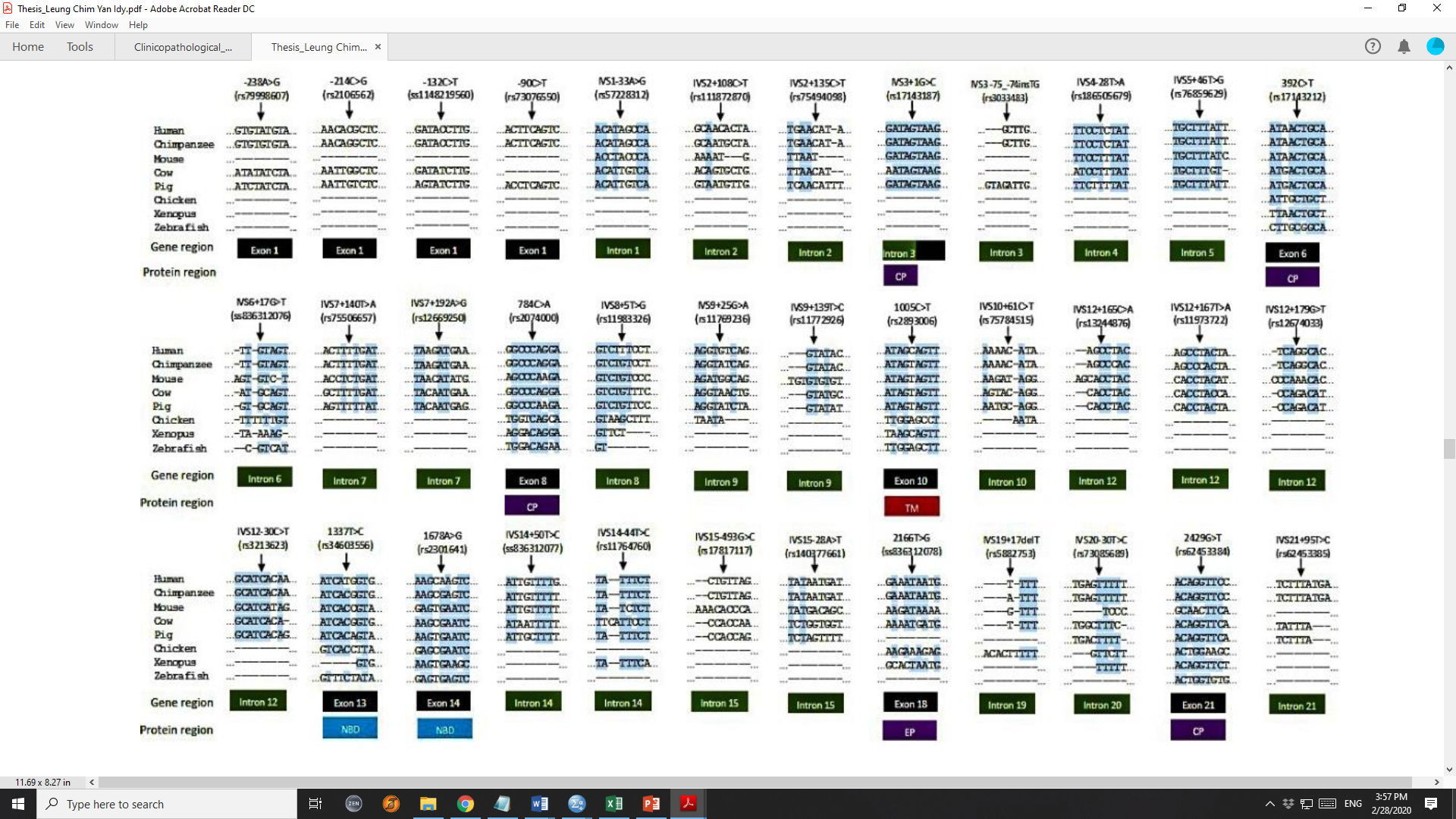
Supplementary Figure 6. Multiple alignment of ABCB5 gene sequence among species.

**Supplementary Table 1. Sequencing platforms used in HCC risk assessment.**

| Order | dbSNP ID | Nucleotide change | Chromosome Position | Sequencing platform |
| --- | --- | --- | --- | --- |
| 1 | rs79998607 | -238A>G | 20615621 | P |
| 2 | rs2106562 | -214C>G | 20615645 | P |
| 3 | ss1148219560^#^ | -132C>T | 20615727 | S |
| 4 | rs73076550 | -90C>T | 20615769 | S |
| 5 | rs57228312 | IVS1-33A>G | 20623232 | S |
| 6 | rs111872870 | IVS2+108C>T | 20623446 | S |
| 7 | rs75494098 | IVS2+135C>T | 20623473 | S |
| 8 | rs17143187 | IVS3+1G>C | 20626612 | S |
| 9 | rs3033483 | IVS3-75_-74insTG | 20628613_20628614 |  |
| 10 | rs186505679 | IVS4-28T>A | 20632031 |  |
| 11 | rs76859629 | IVS5+46T>G | 20632159 | S |
| 12 | rs17143212 | 392C>T | 20643261 | S |
| 13 | ss836312076^#^ | IVS6+17G>T | 20643392 | S |
| 14 | rs75506657 | IVS7 +140T>A | 20643772 |  |
| 15 | rs12669250 | IVS7+192A>G | 20643824 | S |
| 16 | rs2074000 | 784C>A | 20645861 | S |
| 17 | rs11983326 | IVS8+5T>G | 20645885 | S |
| 18 | rs11769236 | IVS9+25G>A | 20646163 | S |
| 19 | rs11772926 | IVS9+139T>C | 20646277 | S |
| 20 | rs2893006 | 1005C>T | 20647558 | S |
| 21 | rs75784515 | IVS10+61C>T | 20647709 |  |
| 22 | rs13244876 | IVS12+165C>A | 20650312 |  |
| 23 | rs11973722 | IVS12+167T>A | 20650314 |  |
| 24 | rs12674033 | IVS12+179G>T | 20650326 |  |
| 25 | rs3213623 | IVS12-30C>T | 20651390 |  |
| 26 | rs34603556 | 1337T>C | 20651424 | S |
| 27 | rs2301641 | 1678A>G | 20658647 | S |
| 28 | ss836312077^#^ | IVS14+50T>C | 20658726 | S |
| 29 | rs11764760 | IVS14-44T>C | 20681461 |  |
| 30 | rs140377661 | IVS15-28A>T | 20685668 |  |
| 31 | ss836312078^#^ | 2166T>G | 20699836 | S |
| 32 | rs5882753 | IVS19+17delT | 20700152 |  |
| 33 | rs73085689 | IVS20-30T>C | 20722986 |  |
| 34 | rs62453384 | 2429G>T | 20723023 | S |
| 35 | rs62453385 | IVS21+95T>C | 20723314 |  |
| 36 | rs4721940 | IVS22-123C>T | 20728192 | P |
| 37 | rs10254317 | 2802G>A | 20728390 | S |
| 38 | rs6461515 | 2908G>A | 20739023 | S |
| 39 | rs6461516 | IVS24+11C>G | 20739150 |  |
| 40 | rs11380694 | IVS24+82_+ 83insG | 20739221_20739222 |  |
| 41 | rs12669866 | IVS24-26T>C | 20742851 | S |
| 42 | rs146201784 | 3042delT | 20742894 | S |
| 43 | rs200759253 | 3547A>C | 20753477 | S |
| 44 | rs189467333 | 4103G>C | 20755953 | S |
| 45 | rs182002068 | 4339G>A | 20756189 | S |
| 46 | ss836312079^#^ | 4609A>G | 20756459 | P |
| 47 | rs12112555 | 4671G>T | 20756521 | S |
| 48 | rs150442227 | 4904G>C | 20756754 | S |
| 49 | rs138210219 | 4964C>G | 20756814 | S |
| 50 | rs3210441 | 5014G>A | 20756864 | S |
| 51 | rs966717 | 5112T>C | 20756962 | S |
| 52 | rs17817117 | IVS15-493G>C | 20685203 | S |
| 53 | rs10231520 | IVS24-406C>T | 20742471 | S |
| 54 | rs10258121 | G>A | 20759290 | S |

Abbreviations: S, SNP genotyping; P, Pyrosequencing

Note: Genetic variants 1-51 were identified in pilot study based on Hong Kong HCC cohort. Genetic variants 52-53 were reported to be significantly associated with reduced melanoma risks, and genetic variant 54 was one of the 44 tagging SNPs of ABCB5 in the same melanoma study (Lin et al., 2013). Chromosome position of SNPs or INDELs referred to the position at chromosome 7 of NCBI human reference genome (GRCh38.p2).

^#^ Novel SNPs. NCBI dbSNP has recoded ss1148219560 to rs554561593, ss836312076 to rs869245984, ss836312077 to rs747900970, ss836312078 to rs869152765, and ss836312079 to rs773871763.

**Supplementary Table 2. Genetic variants associated with HCC risk.**

rs75494098 (IVS2+135C>T)

| Genotype | HCC (n=280) | Control (n=289) | OR (95%CI) | P value |
| --- | --- | --- | --- | --- |
| CC | 274 (97.9%) | 271 (93.8%) | 1.000 (Ref.) | 0.042 |
| CT | 5 (1.8%) | 18 (6.2%) | 0.275 (0.10-0.75) | 0.012 |
| TT | 1 (0.4%) | 0 (0%) | 1.60 x 10^9^ (0) | 1.000 |

rs4721940 (IVS22-123C>T)

| Genotype | HCC (n=299) | Control (n=299) | OR (95%CI) | P value |
| --- | --- | --- | --- | --- |
| CC | 163 (54.5%) | 163 (54.5%) | 1.000 (Ref.) | 0.022 |
| CT | 126 (42.1%) | 110 (36.8%) | 1.145 (0.82-1.60) | 0.428 |
| TT | 10 (3.3%) | 26 (8.7%) | 0.385 (0.18-0.82) | 0.014 |

rs10254317 (2802G>A)

| Genotype | HCC (n=296) | Control (n=299) | OR (95%CI) | P value |
| --- | --- | --- | --- | --- |
| AA | 134 (45.3%) | 136 (45.5%) | 1.000 (Ref.) | 0.036 |
| GA | 143 (48.3%) | 126 (42.1%) | 1.152 (0.82-1.62) | 0.412 |
| GG | 19 (6.4%) | 37 (12.4%) | 0.521 (0.29-0.95) | 0.034 |

Abbreviation: OR: odds ratios; CI: confidence intervals

**Supplementary Table 3. Genetic variants associated with tumor size (n=295).**

|  |  | Tumor size | | P^ | |
| --- | --- | --- | --- | --- | --- |
| Genetic variants | Genotype* | ≤3cm | >3cm |  |  |
| rs73076550 (-90C>T) | CC | 66 | 152 | 0.013 | |
|  | CT | 8 | 57 |  | |
|  | TT | 4 | 8 |  | |
| rs75494098 (IVS2+135C>T) | CC | 67 | 203 | 0.016^#^ | |
|  | CT | 4 | 1 |  | |
|  | TT | 0 | 1 |  | |
| rs76859629 (IVS5+46T>G) | TT | 67 | 203 | 0.047^#^ | |
|  | GT | 11 | 12 |  | |
|  | GG | 0 | 1 |  | |
| rs12669250 (IVS7+192A>G) | AA | 66 | 152 | 0.020 | |
|  | AG | 8 | 55 |  |  |
|  | GG | 4 | 9 |  |  |

^ P value of χ^2^ test, except ^#^ P value of Fisher’s exact test as appropriate.

**Supplementary Table 4. Regression analysis for genetic variants associated with tumor size (n=295).**

|  |  | Tumor size | |  |  |
| --- | --- | --- | --- | --- | --- |
| Genetic variants | Genotype* | ≤3cm | >3cm | OR (95%CI) | P value |
| rs73076550 (-90C>T) | CC | 66 | 152 | 1.000 (Ref.) |  |
|  | CT / TT | 12 | 65 | 2.352 (1.19-4.64) | 0.014 |
| rs75494098 (IVS2+135C>T) | CC | 67 | 203 | 1.000 (Ref.) |  |
|  | CT / TT | 4 | 2 | 0.165 (0.03-0.92) | 0.040 |
| rs76859629 (IVS5+46T>G) | TT | 67 | 203 | 1.000 (Ref.) |  |
|  | GT / GG | 11 | 13 | 0.390 (0.17-0.91) | 0.030 |
| rs12669250 (IVS7+192A>G) | AA | 66 | 152 | 1.000 (Ref.) |  |
|  | AG / GG | 12 | 64 | 2.316 (1.17-4.58) | 0.016 |

**Supplementary Table 5. Genetic variants associated with tumor stage (n=295).**

|  |  | Tumor stage | | P^ |
| --- | --- | --- | --- | --- |
| Genetic variants | Genotype* | Early Stage | Late Stage |  |
| rs2106562 (-214C>G) | GG | 165 | 68 | 0.004^#^ |
|  | GC | 28 | 28 |  |
|  | CC | 3 | 0 |  |
| rs17143187 (IVS3+1G>C) | GG | 42 | 40 | 0.001 |
|  | GC | 102 | 39 |  |
|  | CC | 52 | 17 |  |
| rs17143212 (392C>T) | CC | 165 | 72 | 0.042^#^ |
|  | CT | 28 | 24 |  |
|  | TT | 3 | 0 |  |
| rs2074000 (784C>A) | CC | 42 | 42 | <0.001 |
|  | CA | 101 | 37 |  |
|  | AA | 53 | 17 |  |
| rs10254317 (2802G>A) | AA | 77 | 52 | 0.017 |
|  | AG | 100 | 42 |  |
|  | GG | 17 | 2 |  |

^ P value of χ^2^ test, except ^#^ P value of Fisher’s exact test as appropriate.

**Supplementary Table 6. Regression analysis for genetic variants associated with tumor stage (n=295).**

|  |  | Tumor stage | |  |  |
| --- | --- | --- | --- | --- | --- |
| Genetic variants | Genotype* | Early Stage | Late Stage | OR (95%CI) | P value |
| rs2106562 (-214C>G) | GG | 165 | 68 | 1.000 (Ref.) |  |
|  | CC / CG | 31 | 28 | 2.192 (1.22-3.93) | 0.008 |
| rs17143187 (IVS3+1G>C) | GG | 42 | 40 | 2.619 (1.54-4.45) | <0.001 |
|  | GC / CC | 154 | 56 | 1.000 (Ref.) |  |
| rs17143212 (392C>T) | CC | 165 | 72 | 1.000 (Ref.) |  |
|  | CT / TT | 31 | 24 | 1.774 (0.97-3.23) | 0.061 |
| rs2074000 (784C>A) | CC | 42 | 42 | 2.852 (1.68-4.84) | <0.001 |
|  | CA / AA | 155 | 54 | 1.000 (Ref.) |  |
| rs10254317 (2802G>A) | AA / AG | 177 | 94 | 1.000 (Ref.) |  |
|  | GG | 17 | 2 | 0.222 (0.50-0.98) | 0.047 |

**Supplementary Table 7. Genetic variants associated with tumor nodules (n=295).**

|  |  | Tumor nodules | | P^ |
| --- | --- | --- | --- | --- |
| Genetic variants | Genotype* | One | Multiple |  |
| rs17143187 (IVS3+1G>C) | GG | 48 | 35 | 0.045 |
|  | GC | 105 | 38 |  |
|  | CC | 49 | 20 |  |
| rs17143212 (392C>T) | CC | 172 | 68 | 0.035^#^ |
|  | CT | 28 | 24 |  |
|  | TT | 2 | 1 |  |
| rs2074000 (784C>A) | CC | 49 | 36 | 0.039 |
|  | CA | 102 | 38 |  |
|  | AA | 51 | 19 |  |

^ P value of χ^2^ test, except ^#^ P value of Fisher’s exact test as appropriate.

**Supplementary Table 8. Regression analysis for genetic variants associated with tumor nodules (n=295).**

|  |  | Number of tumor nodules | |  |  |
| --- | --- | --- | --- | --- | --- |
| Genetic variants | Genotype* | One | Multiple | OR (95%CI) | P value |
| rs17143187 (IVS3+1G>C) | GG | 48 | 35 | 1.936 (1.14-3.29) | 0.015 |
|  | GC /CC | 154 | 58 | 1.000 (Ref.) |  |
| rs17143212 (392C>T) | CC | 172 | 68 | 1.000 (Ref.) |  |
|  | CT / TT | 30 | 25 | 2.108 (1.16-3.84) | 0.015 |
| rs2074000 (784C>A) | CC | 49 | 36 | 1.972 (1.16-3.34) | 0.012 |
|  | CA / AA | 153 | 57 | 1.000 (Ref.) |  |
